# Dynamic Monitoring of Changes in Fecal Flora of Giant Pandas in Mice: Co-Occurrence Network Reconstruction

**DOI:** 10.1128/spectrum.01991-22

**Published:** 2022-12-06

**Authors:** Baoxing Gan, Ning Sun, Jing Lai, Zhiqiang Wan, Lianxin Li, Yanyan Wang, Yan Zeng, Dong Zeng, Kangcheng Pan, Jing Fang, Gang Shu, Hesong Wang, Jinge Xin, Xueqin Ni

**Affiliations:** a Animal Microecology Institute, College of Veterinary Medicine, Sichuan Agricultural University, Chengdu, Sichuan, China; b Department of Basic Veterinary, College of Veterinary Medicine, Sichuan Agricultural University, Chengdu, China; c Department of Pharmacy, College of Veterinary Medicine, College of Veterinary Medicine, Sichuan Agricultural University, Chengdu, China; Temasek Life Sciences Laboratory

**Keywords:** giant panda, fecal microbiota transplantation (FMT), gut microbiota, 16S rRNA sequencing, co-occurrence network

## Abstract

Giant pandas are uniquely vulnerable mammals in western China. It is important to develop an animal model to explore the intestinal flora of giant pandas to understand the relationship between digestive diseases and flora. Existing animal models of intestinal flora focus on human flora-associated animals, such as mice, and there is a very limited amount of knowledge regarding giant panda flora-associated animals. To fill this gap, fecal microorganisms from giant pandas were transplanted into pseudosterile and germfree mice using single and multiple gavages. Fecal samples were collected from mice at four time points after transplantation for microbial community analysis. We determined that compared to pseudosterile mice, the characteristics of intestinal flora in pandas were better reproduced in germfree mice. There was no significant difference in microbial diversity between germfree mice and giant panda gut microbes from day 3 to day 21. Germfree mice at the phylum level possessed large amounts of *Firmicutes* and *Proteobacteria*, and at the genus level, Escherichia*-Shigella*, *Clostridium sensu stricto* 1, and Streptococcus dominated the intestinal flora structure. The microbial community co-occurrence network based on indicator species indicated that germfree mice transplanted with fecal bacteria tended to form a microbial community co-occurrence network similar to that of giant pandas, while pseudosterile mice tended to restore the microbial community co-occurrence network originally present in these mice. Our data are helpful for the study of giant panda flora-associated animals and provide new insights for the *in vitro* study of giant panda intestinal flora.

**IMPORTANCE** The giant panda is a unique vulnerable mammal in western China, and its main cause of death is digestive system diseases regardless of whether these animals are in the wild or in captivity. The relationship between the intestinal flora and the host exerts a significant impact on the nutrition and health of the giant pandas. However, the protected status of the giant panda has made *in vivo*, repeatable, and large-sample sampling studies of their intestinal flora difficult. This greatly hinders the research depth of the giant panda intestinal flora from the source. The development and utilization of specific animal models to simulate the structure and characteristics of the intestinal flora provide another means to deal with these research limitations. However, current research examining giant panda flora-associated animals is limited. This study is the first to reveal dynamic changes in the fecal flora of giant pandas in mice after transplantation.

## INTRODUCTION

Giant pandas of the order *Carnivora* are endemic to western China and are regarded as national treasures. Their distinctive black and white coloration and body shape make them widely welcomed worldwide as protected animals (1–3). The unique dietary habits of giant pandas are worth noting, and there have been a large number of studies examining the digestive system of giant pandas (2, 3). The digestive system in the giant panda resembles that of others in the order *Carnivora*, despite the knowledge that its diet is primarily bamboo. For giant pandas, the digestive system characteristics of typical predators include sharp canine molar teeth, short total intestinal tract, absence of cecum structure, and short residence time of the digestive tract (4, 5). The particular nature of the digestive system of giant pandas has attracted the interest of researchers from around the worldwide. Over the past 2 decades, significant advances in molecular biology have led us to determine that the diet of giant pandas was converted to bamboo-based approximately 2 million years ago (6, 7). There is ongoing research regarding the scientific evidence of giant pandas being able to digest cellulose. Publicly reported studies have demonstrated that giant panda genomes lack digestive cellulase genes, suggesting that this metabolism is more likely to be associated with intestinal flora rather than with the digestive system itself (8). Subsequent *in vitro* studies confirmed that *Clostridium* species in the intestinal flora of giant pandas could encode genes related to cellulase, β-glucosidase, 1,4-β-xylanase, and 1,4-β-xylanase (9, 10). It has been reported that the microbial community structure of the giant panda intestinal flora is similar to that of carnivores, as its diversity is extremely low and its *Clostridium* members are not related to cellulose decomposition or flora of herbivores, based on phylogeny (10, 11). Compared to herbivorous animals, the activities of cellulase and hemicellulase in giant panda feces were the lowest. Therefore, it was concluded that cellulose is not the primary energy source of giant pandas, and the intestinal flora is not the key to cellulose decomposition (11). Although it has been revealed that its digestibility is very low, the corresponding reduction in the size of brains, livers, and kidneys and the low physical activity due to increasing feeding time and food intake enables a low level of energy consumption (12). Although controversy regarding the intestinal flora in giant pandas continues, there is no doubt that the intestinal flora is essential for host health. Changes in intestinal flora composition can cause gastrointestinal discomfort, and intestinal disease is the main cause of death in both wild and captive pandas (13–15). For example, giant pandas fed a bamboo diet exhibit mucus fecal excretion symptoms that may be related to the increase in bacterial abundance of *Pasteurellaceae* and a high-protein diet (13). Many studies have been conducted to examine intestinal flora. As previously reported, the dominant phyla of intestinal microorganisms in giant pandas are *Firmicutes* and *Proteobacteria*, and these are primarily Escherichia*-Shigella*, *Clostridium*, Streptococcus, and *Lactococcus* at the genus level (11, 16). Interseasonal variations can cause significant fluctuations in Streptococcus and *Lactobacillus*, but not in Escherichia*-Shigella*, *Clostridium*, or *Turicibacter* (11). Pandas eat different parts of bamboo plants in different seasons. This diet can lead to significant changes in the composition of the intestinal flora of the pandas. The abundance of *Proteobacteria* in the intestinal flora changes as the panda diet changes from eating bamboo shoots to consuming poles. Changes in the abundance of Escherichia coli at the species level are particularly noticeable (17). Increased *Clostridium* and a lack of *Lactobacillaceae* were observed in giant pandas with anorexia; however, the relationship between anorexia and flora change remains unclear (18). To further promote the research and protection of giant pandas that are ancient and precious species and to provide better prevention means for potential digestive system diseases, it is particularly important to study the relationships among the organisms comprising the intestinal microflora.

Unfortunately, as a conservation animal, *in vivo* studies of the intestinal flora of the giant panda are almost impossible. As giant pandas are extremely endangered, destructive sampling is no longer allowed in scientific research activities or disease diagnosis and treatment. Currently, most of the reported samples of intestinal flora from giant pandas are collected from feces and occasionally from pandas that died due to accidental causes (19, 20). The limitation of a single sample source greatly hinders research into changes in the intestinal flora of the giant panda. The development and utilization of animal models to simulate disease symptoms or special phenotypes provide a means to overcome the limitations of current research and could greatly promote the development of medical research (21–23). However, for giant pandas, the lack of animal models for alternative intestinal flora research has greatly hindered further exploration of the interactions between their intestinal flora and host physiological characteristics and the development and application of their intestinal health regulation methods. Fecal microbiota transplantation to simulate the intestinal flora of specific populations or animals is regarded as a new means of intestinal research for specific populations or animals (24, 25). Human intestinal flora have been transplanted into gnotobiotic animals to simulate human microflora and to study host-microbial cross talk and specific microbial functions (26, 27). However, animal models applied to the study of intestinal flora are primarily related to human intestinal flora, while animal models related to pig intestinal flora have rarely been reported (28, 29).

Currently, there are no in-depth studies examining animal models of giant panda flora-associated (GPFA) animals. In this study, fecal microbiota transplantation and 16S rRNA sequencing technology were used to construct intestinal flora-associated mouse models of giant pandas, using germfree mice and pseudosterile mice (antibiotic-treated mice). We aimed to determine if this method could simulate the main characteristics of the intestinal flora of giant pandas and provide more theoretical information to explore the best model establishment method.

## RESULTS

### 16S rRNA gene sequencing results.

All samples were sequenced using the Illumina MiSeq platform. A total of 6,337,424 raw reads were obtained from 107 fecal samples (10 giant panda fecal samples, 1 mixed sample, and 96 mouse fecal samples), with an average of 59,228 raw reads per sample. After quality filtering, denoising, and chimera removal, 3,121,274 amplified sequence variants (ASVs) were accumulated, ultimately resulting in a total of 2,461 ASVs with an average of 107 ASVs per sample. The rarefaction curves determined that the observed species number gradually stabilized, suggesting that the sequencing data were reasonable and that there was a uniform species composition within the sample (see Fig. S1 in the supplemental material). The current results are sufficient to reflect the diversity of the sample, and subsequent analyses can be performed.

### Gut microbiota differs in pandas and mice.

**FIG 1:**
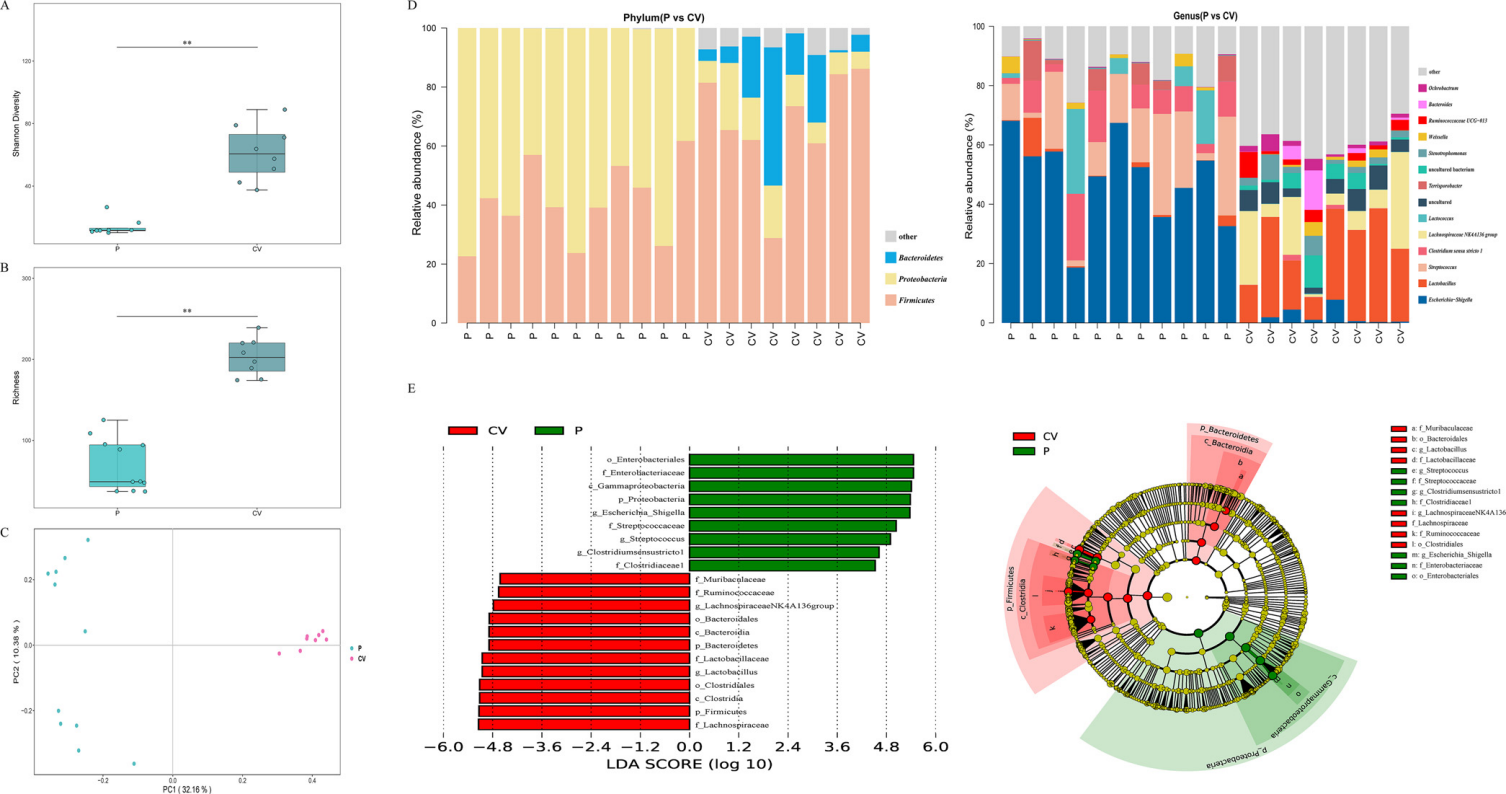
Comparison of gut microbiota between giant pandas and mice. (A) Shannon diversity index analysis boxplots. (B) Richness index analysis boxplots. Statistical differences in both panels were determined by the Kruskal-Wallis test (*, *P* < 0.05; **, *P* < 0.01). All of the data are expressed as means with standard deviations. (C) PCoA analysis of beta diversity based on Bray-Curtis distance. The data were statistically analyzed based on ADONIS. (D) Distributions of relative abundances of top species at the phylum and genus level. Each color represents a species. The height of the column represents the abundance of reads. (E) Taxa that were significantly differentially represented between groups were examined by LEfSe using the parameters LDA value of >4 and *P* value of < 0.05. CV, SPF mice without any treatment; P, giant pandas.

The Shannon diversity index comprehensively reflects community effective number of species. We noted that the alpha diversity index richness and Shannon diversity index of the giant panda group (P group) was significantly lower than that of the mice control group (CV group) (*P* < 0.01) (Fig. 1A and B). Most of the ASVs were assigned to 14 phyla and 160 genera, and in subsequent analysis we classified a relative abundance of <1% for “Others” (Fig. 1D and E, genera and phyla with relative abundance ≥1%). At the phylum level, *Firmicutes* (40.69%, 22.62% to 61.75%) and *Proteobacteria* (55.22%, 38.25% to 77.38%) were the two dominant phyla in the giant panda intestinal flora (percentages in parentheses are expressed as “mean relative abundance”, “minimum relative abundance to maximum relative abundance”). Unlike the P group, in the CV group mouse *Proteobacteria* (11.65%, 7.03% to 22.74%) exhibited lower abundance, and the relative abundances of other phylum levels were higher than was that of the P group, including *Firmicutes* (67.85%, 28.81% to 86.19%) and *Bacteroidetes* (15.05%, 0.77% to 46.83%). At the genus level, Escherichia*-Shigella* (48.97%, 18.68% to 68.13%) was the dominant genus in the P group. Additionally, the relative abundances of Streptococcus (16.68%, 1.71% to 34.07%), *Clostridium sensu stricto* 1 (8.62%, 0.03% to 22.46%), *Lactococcus* (5.47%, 0% to 28.56%), and *Terrisporobacter* (3.7%, 0% to 13.38%) in the P group were significantly higher than were those in the CV group (*P* < 0.01). In contrast to the P group, *Lactobacillus* (24.33%, 7.55% to 38.23%) and *Lachnospiraceae* NK4A136 group (12.28%, 0.85% to 32.54%) were the dominant bacterial genera in the CV group. At the phylum and genus levels, we observed clear differences in the compositions of the CV and P groups (Fig. 1D).

The next section of the survey compared the overall similarities between the two groups. Further principal-coordinate analysis (PCoA) determined that the clusters in the CV group were more concentrated and farther from the P group cluster (Fig. 1C). Similarly, to fully explore the differences in the composition of the intestinal flora between giant pandas and mice, it was also important to assess the specific taxonomic differences between the two groups. Pairwise comparisons between the two groups were performed using linear discriminant analysis effect size (LEfSe). The characteristics of these comparisons are presented in Fig. 1E, where we indicate the differential flora with an linear discriminant analysis (LDA) value of >4. At the genus level, Escherichia-*Shigella*, *Clostridium sensu stricto* 1, and Streptococcus were significantly higher in the P group than they were in the CV group (*P* < 0.05) (Fig. S2A). Similarly, we also observed that *Lactobacillus* and *Lachnospiraceae* NK14A136 group were significantly higher in the CV group (*P* < 0.05) (Fig. S2B).

### Dynamic changes of fecal flora of giant panda transplanted into mice.

**FIG 2:**
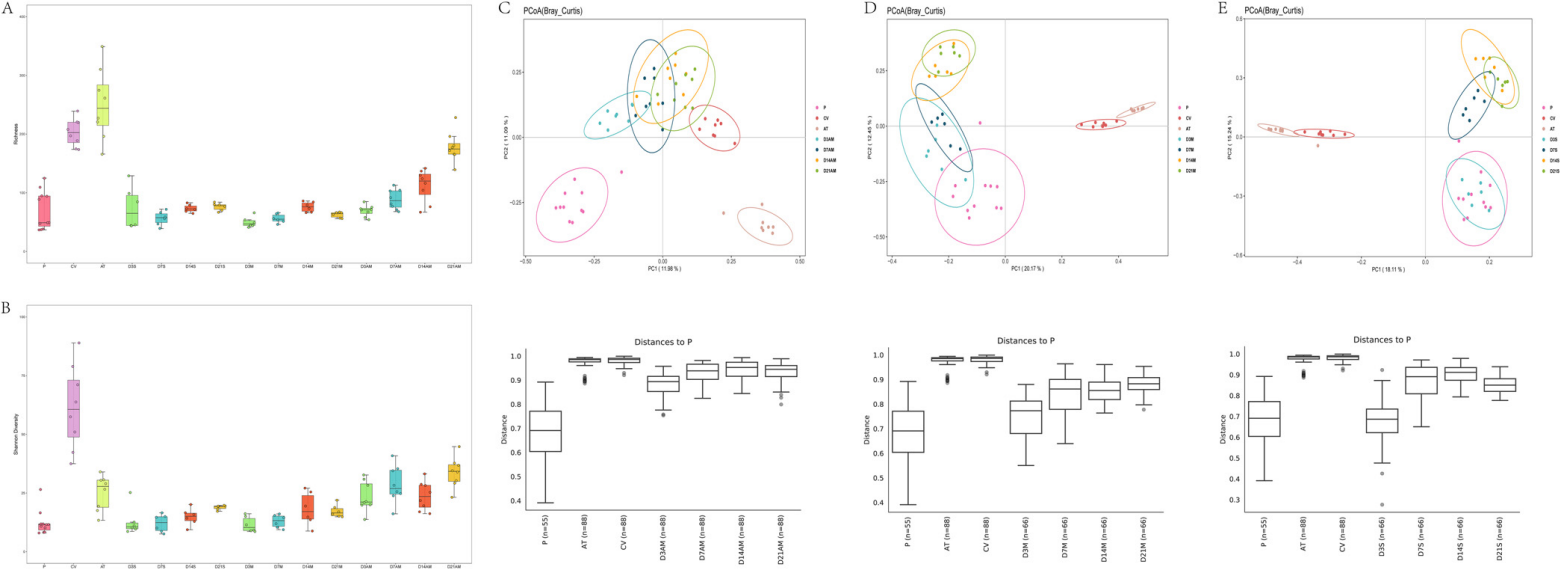
Dynamic changes in fecal flora of giant panda transplanted into mice. (A) Richness index analysis boxplots. (B) Shannon diversity index analysis boxplots. (C) PCoA analysis of AM group beta diversity based on Bray-Curtis distance. (D) PCoA analysis of M group beta diversity based on Bray-Curtis distance. (E) PCoA analysis of S group beta diversity based on Bray-Curtis distance. A-B statistical differences were determined by the Kruskal-Wallis test (A and B) or permutational multivariate analysis of variance (C to E) (*, *P* < 0.05; **, *P* < 0.01). Results of each group comparison are provided in Table S2 in the supplemental material. CV, SPF mice without any treatment; P, giant pandas; AT, SPF mice treated with antibiotics; AM, SPF mice treated with antibiotics and then received multiple fecal microbiota transplants; M, germfree mice treated with multiple fecal microbiota transplants; S, germfree mice treated with single fecal microbiota transplants and sampled at day 3, 7, 14, and 21 posttransplantation.

Previously providing mice with drinking water supplemented with antibiotics resulted in two treatment groups that included the antibiotic treatment group (AT) and the multiple fecal microbiota transplantation group (AM). Antibiotic treatment significantly reduced the Shannon diversity index (*P* < 0.01) (Fig. 2B, Table S2), and we noticed an abnormal increase in the richness index in the AT group (Fig. 2A). Compared to the CV group, the AM alpha diversity index remained at a low level, and its richness index was significantly different (*P* < 0.01) with the exception of the richness index at day 21, which was not significantly different (Fig. 2A and B, Table S2). As presented in Fig. 2C, the P, AT, and CV groups were clustered into one cluster and were far apart (Table S3). The results of the AM group on day 3 were closest to those of group P, and the similarity was the highest; however, as time elapsed, the AM group clusters began to trend toward the position of the CV group. We then analyzed the microbial composition (Fig. S3). At the phylum level, antibiotic treatment disturbed the composition of the intestinal flora in the AT group. Compared to the CV group, the relative abundances of *Firmicutes* (21.00%, 8.65% to 45.79%), *Bacteroidetes* (1.34%, 0.84% to 2.29%), and *Verrucomicrobia* (0.01%, 0% to 0.047%) in the AT group were significantly reduced (*P* < 0.05), while *Proteobacteria* (65.98%, 50.50% to 76.26%) was increased significantly (*P* < 0.05). At the genus level, antibiotic treatment significantly reduced (*P* < 0.05) the relative abundances of numerous genera (*Lactobacillus*, *Lachnoclostridium*, *Bacteroides*, *Akkermansia*, *Parabacteroides*, *Blautia*, and *Lachnospiraceae* NK4A136 group), and this caused the composition of the AT and CV groups to be different. In the AM group, the relative abundance of *Proteobacteria* (21.72% to 7.91%) and *Bacteroidetes* (43.84% to 12.51%) decreased significantly (*P* < 0.05) and stabilized over time, while that of *Firmicutes* (32.27% to 66.16%) increased significantly (*P* < 0.05) with greater fluctuations. Additionally, we also observed that *Verrucomicrobia* (1.78% to 21.28%) was more abundant in the AM group, while the relative abundance in the CV and AT groups was <0.20%. In the AM group, the results on days 3 and 7 indicated that the abundances of the *Lachnospiraceae* NK4A136 group and *Turicibacter* were colonized, but as time elapsed, they gradually decreased in stability. In particular, Escherichia*-Shigella*, *Bacteroides*, *Parabacteroides*, and Klebsiella decreased significantly (*P* < 0.05) from day 3 to 21. We also observed that *Lachnoclostridium* (3.99% to 4.70%) exhibited a very stable abundance from day 7.

Two groups (M and S) were produced by single and multiple fecal microbiota transplantation (FMT) of giant panda fecal flora into germfree mice. We dynamically observed changes in the flora of germfree mice after FMT by sequencing. With the passage of implantation time, the richness of the M and S groups fluctuated but was always lower than that of the P and AM groups. There was no significant difference between the M and P group from day 3 to 21 (Fig. 2A, Table S2). Shannon diversity index analysis indicated that after transplantation, the M and S groups exhibited an upward trend that was only significantly different (*P* < 0.05) from that of the P group at 21 days (Fig. 2B, Table S2). Most ASVs were assigned to 10 phyla and 80 genera. At the phylum level (Fig. S3A), the dominant phyla *Firmicutes* and *Proteobacteria* in the P group were colonized in germfree mice; however, the abundance of *Firmicutes* in the M and S groups was higher. At the genus level, we observed in detail the changes in species over time in germfree mice. The species abundance and composition of germfree mice in the M and S groups and the P group differed at the genus level (Fig. S3B). From the perspective of the main bacterial genera, in the germfree mouse samples on days 3 and 7 the genera Escherichia*-Shigella* (7.01% to 50.94%), *Clostridium sensu stricto* 1 (11.72% to 42.04%), and Streptococcus (0.24% to 49.67%) were dominant, and this was consistent with the trend of the donor P group. Overtime, the abundance of some bacterial genera in the M and S groups changed to varying degrees and stabilized after day 14. It was clearly observed that, from day 7, the abundance of Streptococcus (the main genus of bacteria) in germfree mice began to decrease. Concurrently, the abundance of low-abundance genera in the P group, such as *Lachnoclostridium*, *Lactobacillus*, *Turicibacter*, *Stenotrophomonas*, *Ochrobactrum*, Klebsiella, *Blautia*, and *Enterococcus*, increased and stabilized in the M and S groups. The abundance of *Enterococcus* decreased significantly from day 3 to day 21, while that of *Lachnoclostridium* and *Turicibacter* increased significantly (*P* < 0.05). The results of the PCoA analysis indicated that the P and AT groups were distributed in different locations and were far away, indicating that the similarity between the two groups was lower than that of the other groups (Fig. 2, Table S3). Moreover, the giant panda flora-associated mice after multiple or single gavage treatment could be gathered in one well, and the difference between the groups was not obvious. At days 3 and 7 after the implantation of the flora, the distance between the M and S groups of mouse flora and the giant panda flora P was relatively close (Table S3). Among them, it is worth noting that the intestinal flora of the S group and the giant panda on day 3 exhibited the best similarity, and this almost completely overlapped with the P in the PCoA graph.

### Retention of community network characteristics of giant panda intestinal flora in mice.

**FIG 3:**
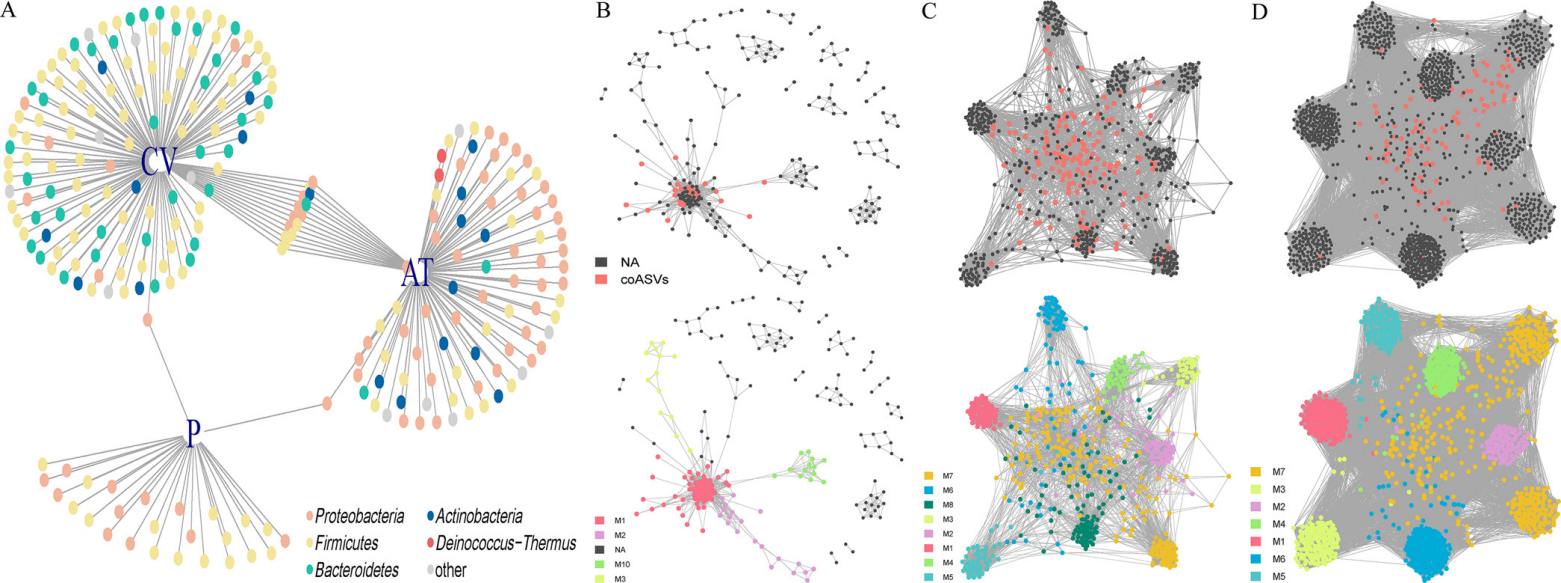
Intestinal flora network and module identification for giant pandas and mice. (A) Bipartite networks of indicator species analysis. Different group-specific ASVs in the bacterial communities are displayed, based on indicator species analysis. Circles represent individual ASVs that are positively and significantly associated (*P* < 0.01) with one or more different grouping factors (associations given by connecting lines). ASVs are colored according to their phylum assignment. (B) Co-occurrence network based on indication species display with P group bacteria network (*r* > 0.3, *P* < 0.05). The intersection analysis of the indicator species shows ASVs included in the SparCC co-occurrence network, retaining a network of indicator species and their associated species shared by both. The colors of ASVs are divided into modules based on the communication relationship with the indicator species ASVs. Each node represents an ASV, and gray edges represent the communication of ASVs. M is shorthand for a module, and the number simply indicates that it is a different module. NA refers to the species that may be associated with the indicator species in the network but are classified as not belonging to any module in the module identification. In this study, we defined ASVs in the M group as key species and those in the NA group as peripheral species. (C and D) Similar to the results shown in panel B, showing the CV and AT group co-occurrence network.

The interaction of the intestinal flora is very important for the stability of healthy biological communities. Therefore, we explored the bacterial community co-occurrence network in each group. By comparing the ASVs among the different groups, the indicator species in each group used in this study were determined according to a *P* value of <0.01 (marked as red dots in the co-occurrence network). There were different numbers and categories of indicator species between different groups, and these indicator species could represent the main characteristics of their intestinal flora. A few bacterial ASVs shared between the CV and AT groups reflected similar bacterial communities (Fig. 3A). However, the P group possessed only one shared indicator species, compared to the other groups, thus indicating that the P group possessed more unique and easily distinguished indicator species (Fig. 3A). We determined the indicator species of each group on the co-occurrence network and then identified the bacterial community communication module related to the indicator species. For group P, we identified 24 indicator species that formed four different modules in the subsequent module division (Fig. 3B, Table S1). The difference was that the numbers of indicator species, nodes, and edges in the intestinal flora network of the P group were much smaller than those for the CV and AT groups, indicating that the interaction between the giant panda intestinal microorganisms was lower, the network structure was simpler, and the internal community was smaller. For CV, there were 141 indicative species, and eight modules were formed. The number of ASVs and the first three modules with rich communication with the indicative species were M7, M6, and M8 (Fig. 3C, Table S1). Similarly, in the AT group, the number of indicated species was lower than that in the CV group due to the impact of antibiotics. This group did exhibit 1,263 nodes and 74,446 edges in the network that were divided into seven modules (Fig. 3D, Table S1). The first three modules were M7, M3, and M2.

**FIG 4:**
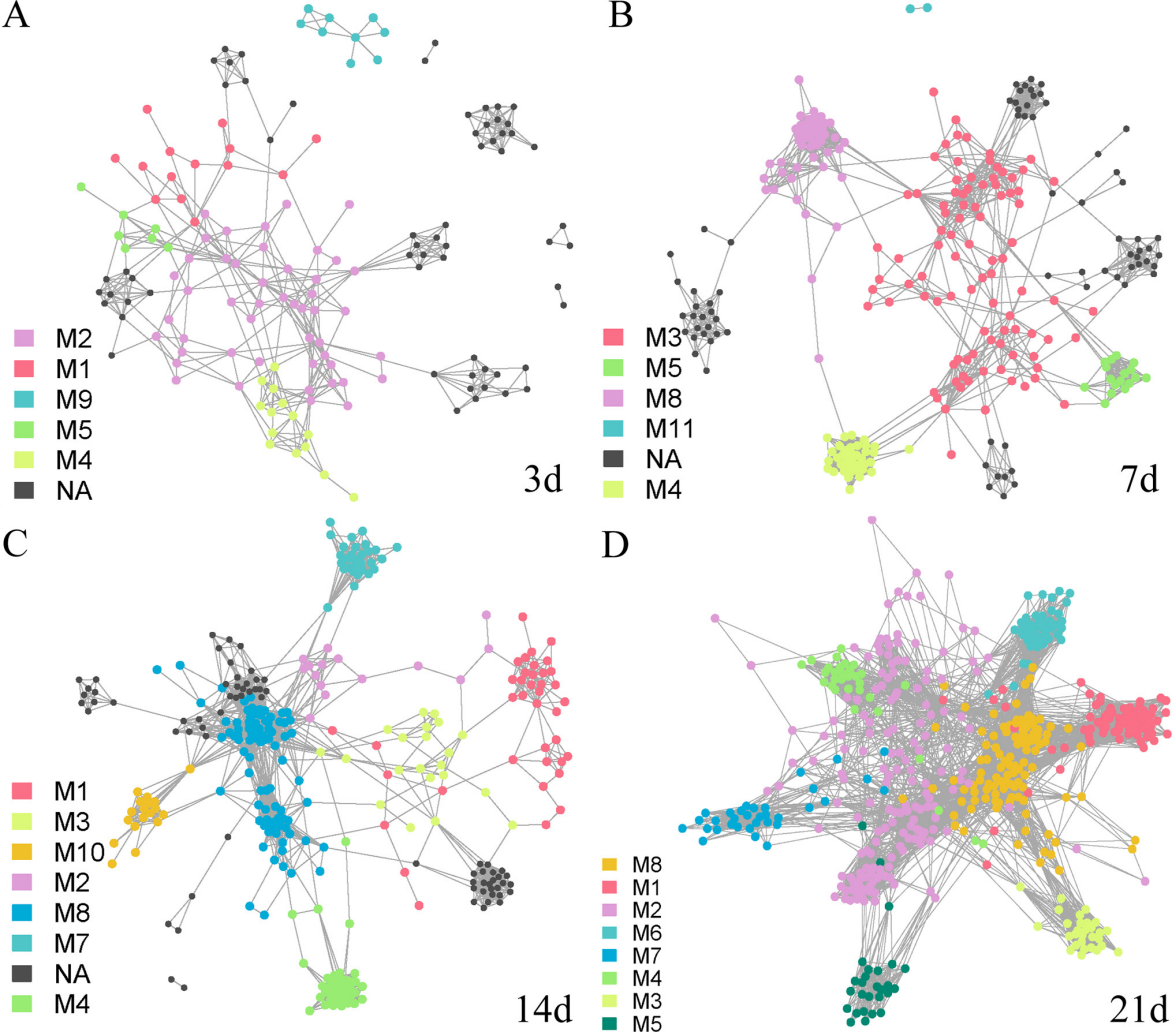
Co-occurrence network of intestinal flora in the AM group at different time points. The co-occurrence network based on indicated species display the AM group bacteria network (*r* > 0.3, *P* < 0.05). The colors of ASVs are divided into modules based on the communication relationship with the indicator species ASV. (A, B, C, and D) Data from day 3, 7, 14, and 21, respectively. The meanings of nodes, edges, M, and NA are as described previously.

For the AM group, we determined that the characteristics of the co-occurrence network at day 3 were most similar to those of the donor P group, and the topological structure parameters were also very similar (Fig. 4A, Fig. S4, Table S1). In the following time, the number of ASV nodes in the co-occurrence network increased, and the original module composition structure was changed. Many ASVs that did not belong to any module were defined as NA (Fig. 4B and C, Fig. S4, Table S1). On day 21, we observed that the network structure tended toward the original network structure of mice, although they differed in module division and composition (Fig. 3C, Fig. 4D). This suggested that antibiotic-treated mice did not retain the characteristics of the co-occurrence network in the P group. The interaction between the original bacteria that could not be eliminated by antibiotics in mice and the giant panda intestinal flora reversed the flora network of the AM group from day 3 to day 21.

**FIG 5:**
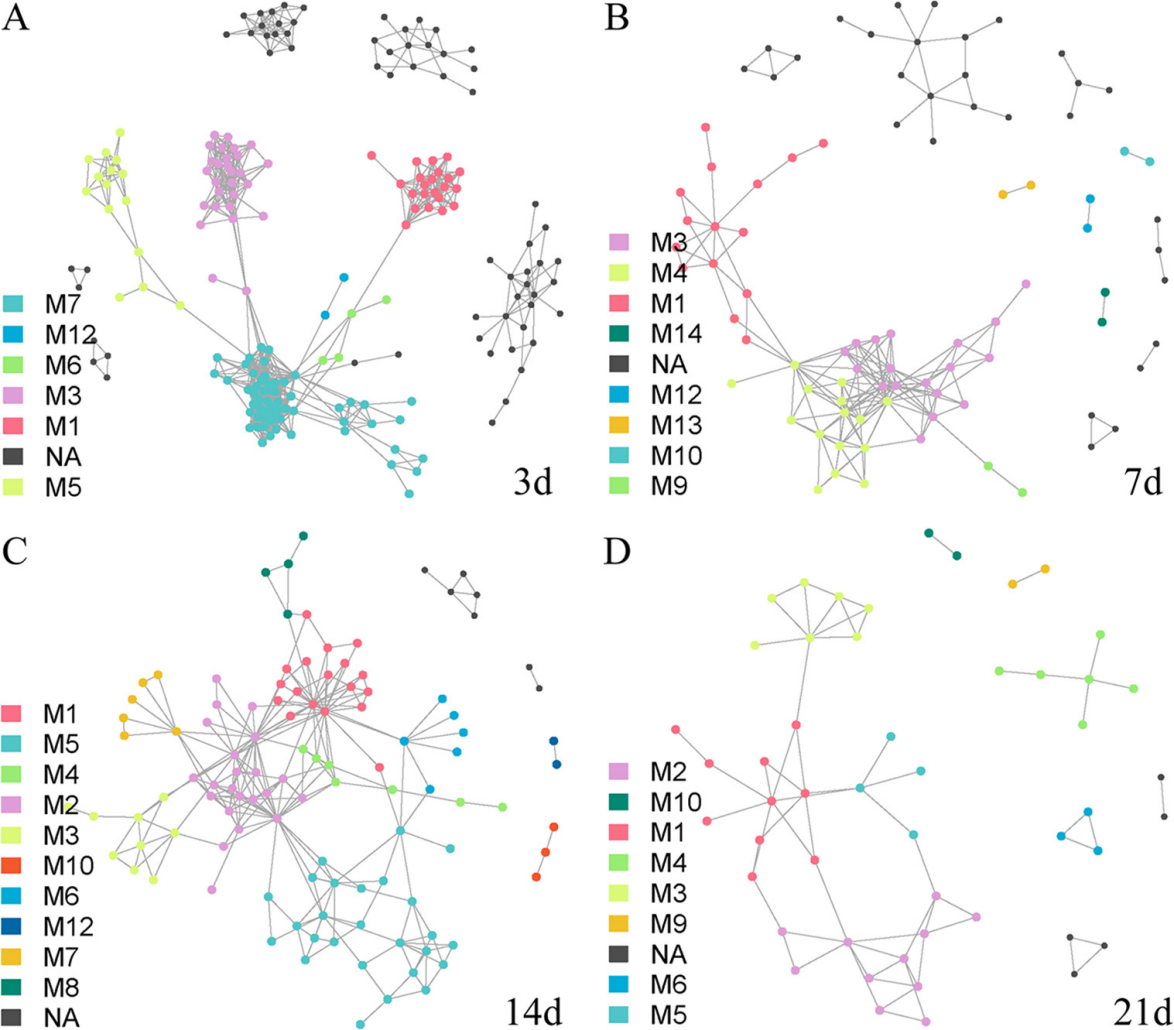
Co-occurrence network of intestinal flora in the S group at different time points, based on indication species displayed in the S group bacteria network (*r* > 0.3, *P* < 0.05). The colors of ASVs are divided into modules based on the communication relationship with the indicator species ASV. (A, B, C, and D) Data from day 3, 7, 14, and 21, respectively. The meanings of nodes, edges, M, and NA are as described previously.

**FIG 6:**
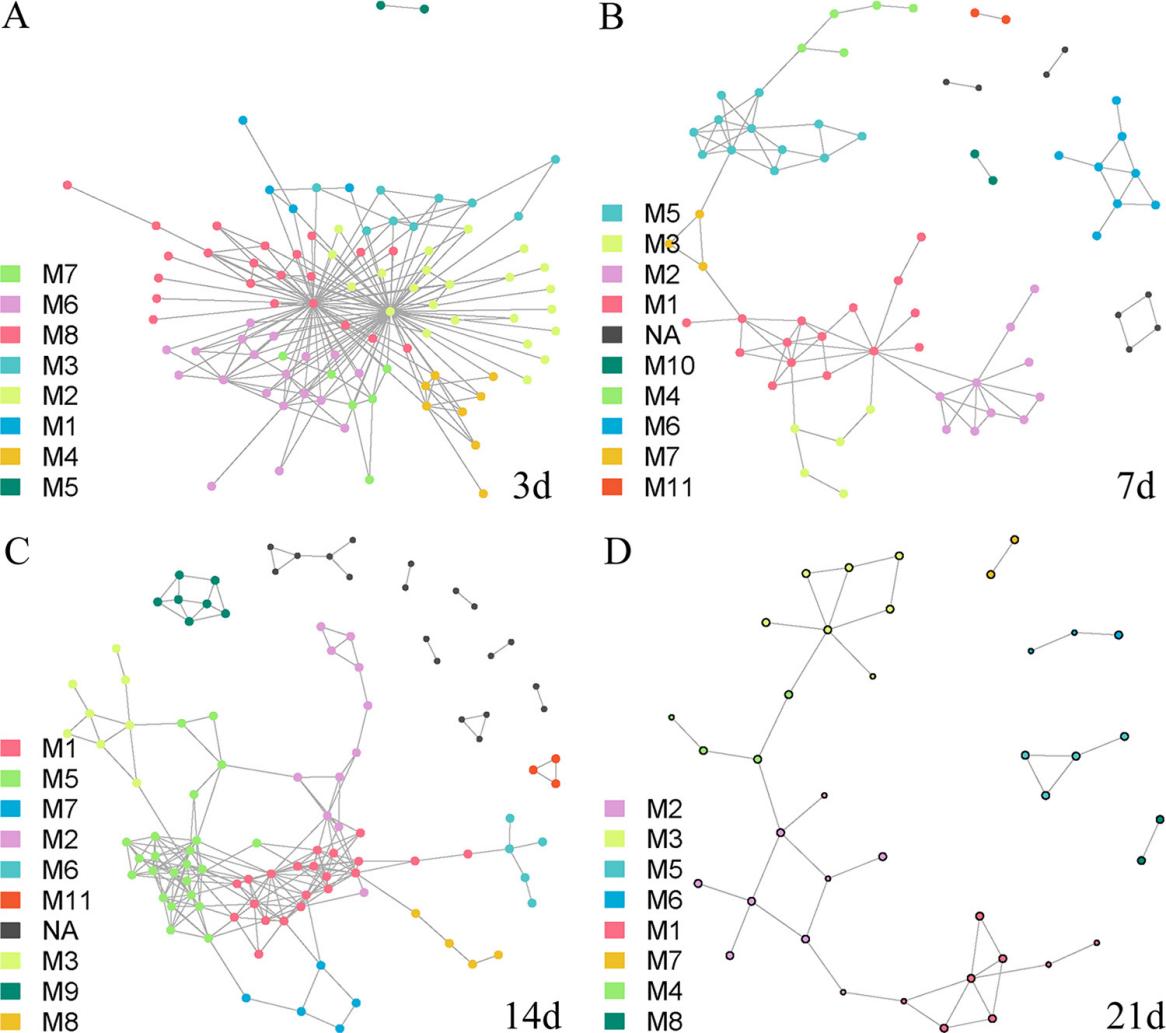
Co-occurrence network of intestinal flora in the M group at different time points, based on indication species displayed in the M group bacteria network (*r* > 0.3, *P* < 0.05). The colors of ASVs are divided into modules based on the communication relationship with the indicator species ASVs. (A, B, C, and D) Data from day 3, 7, 14, and 21, respectively. The meanings of nodes, edges, M, and NA are as described previously.

Neither group M nor group S exhibited a structure of the complex bacterial community communication networks similar to that for mice. For the S group, the flora of the P group with a single inoculation of donor changed from days 3 to 21, and the module structure, nodes, and edge numbers were similar to those of the P group (Fig. 5, Fig. S6, Table S1). However, it is worth noting that ASV nodes above the threshold in the network decreased, suggesting that many bacterial interactions in mice became weaker. In contrast, the M group with multiple gavages from the P group did not exhibit the network characteristic structure of the P group on day 3, but it gradually exhibited a network characteristic similar to that of the donor P group in the subsequent sampling (Fig. 6, Fig. S5, Table S1). Similar to the S group, the communication between many ASV nodes gradually weakened, and the number of ASV nodes in the module decreased. These results indicated that the intestinal flora in the M and S groups retained the main characteristics of the flora network from the intestinal flora of giant pandas. This inference was further supported by subsequent analyses.

**FIG 7:**
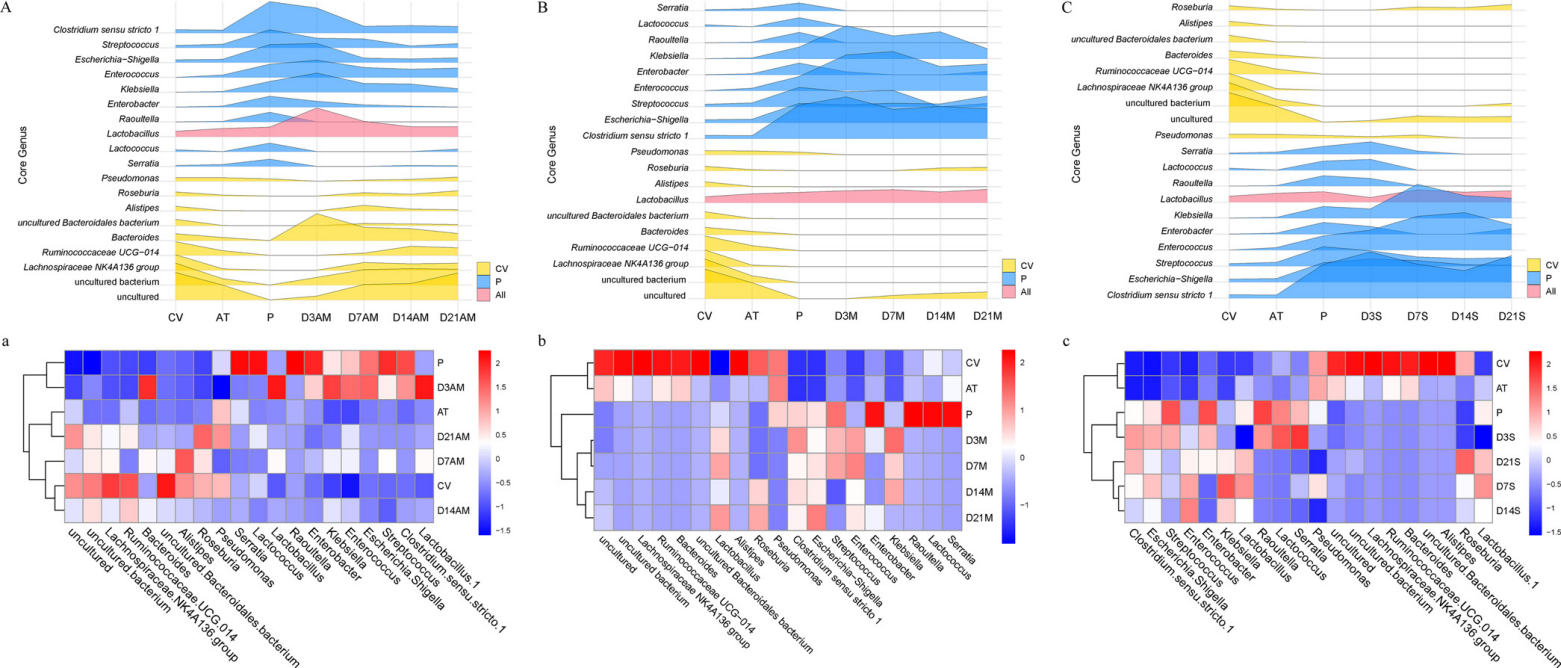
Changes in the species number for the genus-level ASVs in each group. The ASV species (not their abundance) in the genus taxa of giant pandas and mice were statistically analyzed. Here, we focused on the top 10 taxonomic genera of the statistical results. Peak maps A, B, and C show the number changes of these genera in the AM, M, and S groups (CV group is marked in yellow, P group is marked in blue, common genera are marked in pink). Heatmaps a, b, and c show the clustering relationships between the changes of these genera in the AM, M, and S groups and P, CV, and AT groups.

The ASVs in the modules of each group of co-occurrence networks were extracted, and species annotation and classification statistics were performed. After annotating the ASV species contained in the co-occurrence network module of group P, we obtained the top 10 species (Klebsiella, *Enterococcus*, Enterobacter, Escherichia*-Shigella*, Streptococcus, *Clostridium sensu stricto* 1, *Lactobacillus*, *Raoultella*, *Lactococcus*, and *Serratia*) according to classifications at the genus level (not relative abundance), and we observed the classification of these 10 genera in the co-occurrence network of fecal microbiota transplantation mice. Using the same method, we also obtained the top 10 genera of the CV group, which included *Lactobacillus*, Pseudomonas, *Roseburia*, *Alistipes*, *Bacteroidales bacterium*, *Bacteroides*, *Ruminococcaceae* UCG-014, *Lachnospiraceae* NK4A136 group, uncultured bacterium and uncultured. Figure 7A indicates that the number of Klebsiella, *Enterococcus*, Escherichia*-Shigella*, Streptococcus, *Clostridium sensu stricto* 1, and *Lactobacillus* taxa decreased and approached 0 (*Raoultella*, *Lactococcus*, and *Serratia*) from day 7 in group AM. The number of taxa belonging to the CV group (uncultured, uncultured bacterium, *Lachnospiraceae* NK4A136 group, *Ruminococcaceae* UCG-014, *Bacteroides*, uncultured *Bacteroidales bacterium*, *Alistipes*, and *Roseburia*) increased and stabilized in group AM. These genera were similar to those of the P group at day 3 in the AM group and were clustered together (Fig. 7a). Subsequent analyses for days 7, 14, and 21 revealed that this group clustered together with CV and AT. This indicated that the AM group could retain these major genera from the P group after 3 days and were more similar to mice in subsequent developments. This was different from the P group. In group M, *Raoultella*, *Lactococcus*, and *Serratia* from group P were lost after 3 days, while other genera were colonized and stabilized (Fig. 7B). Pseudomonas and uncultured bacteria originally belonging to group P were enriched and stable in group M mice. The heatmaps revealed that the day 3, 7, 14, and 21 features of M were clustered together and were similar to those of the P group, but different from those of CV and AT (Fig. 7b). In group S, 10 taxa from group P maintained a trend that was similar to that of group P. After 7 days, *Raoultella*, *Lactococcus*, *Serratia*, and Enterobacter decreased gradually, and this was accompanied by an increase in species belonging to specific taxa in the P group (*Roseburia*, uncultured, and uncultured bacterium) (Fig. 7C). Furthermore, P and the day 3 S group were clustered directly into the highest similarity group, and this was followed by the day 7, day 14, and day 21 data for the S group (Fig. 7c). This indicated that regardless of the M or S group, fecal microbiota transplantation could retain the characteristics of these genera from the intestinal flora of the giant pandas. In particular, the day 3 S group was the most similar to that of the P group, but the similarity to the characteristics of giant panda flora changed over time. This result was consistent with the previous co-occurrence network analysis (Fig. 5).

## DISCUSSION

To establish a mouse model of giant panda flora-associated (GPFA) that is highly similar to the intestinal physiology and metabolic characteristics of giant pandas, it is necessary that the composition and structure of the intestinal flora of mice be similar to that of giant pandas. Therefore, mastering the basic composition and characteristics of intestinal flora and understanding intestinal flora are the bases for the in-depth study of the interaction mechanism between intestinal flora and the host, and these are also the key problems to be solved in the establishment of GPFA mouse models. Constructing a stable model for studying animal gastrointestinal diseases using germfree mice has become an important scientific research method in recent years (22, 30, 31). Previous studies reported that the use of fecal bacteria from overweight and thin individuals could reproduce human phenotypes in germfree mice, thus paving the way for the study of the mechanistic link between flora and human phenotypes (21). Currently, human flora-associated rat, pig, dog, and cattle models have established that mouse models are still the first choice for the vast majority of microbial genomics research projects (32, 33). The composition of the intestinal flora in mice and humans is significantly different. However, the use of germfree mice to establish human intestinal flora transplantation mouse models and to study the influence of intestinal flora on human health and related diseases has become an important research strategy and design idea in the current research field. Concurrently, due to the large proportion of giant panda deaths caused by digestive system diseases, clinical research does not allow traumatic sampling, and this greatly increases the difficulty of research and hinders prevention of giant panda digestive system diseases (14). Regardless of whether it is used for the protection of giant pandas or for nutritional research, there is an urgent need for an alternative research model that can fully or partially represent giant pandas. As mentioned earlier, the composition of intestinal flora is closely related to the host food and phylogenetic status (34). Although the structure of the mouse intestine is different from that of the giant panda, as an omnivorous animal, combined with the technology of fecal bacterial transplantation, it does exhibit the potential to colonize the intestinal flora of the giant panda. Therefore, we used germfree mice, borrowed from the technology of human flora-associated model establishment, and transplanted the fecal bacteria of giant pandas into mice. We used 16S rDNA sequencing technology to quickly compare and analyze the composition and differences in intestinal flora between giant pandas and mice and observed the dynamic changes in the fecal flora of pandas after being transferred into pseudosterile (antibiotic-treated) mice and germfree mice.

According to our study, the diversity and species composition of intestinal flora are significantly different between giant pandas and mice. Consistent with previous studies examining giant panda intestinal flora, in this study we observed that *Proteobacteria* and *Firmicutes* were the dominant phyla in giant pandas, whereas the abundance of other phyla was low (9, 10, 35). Among the top 10 genus genera in terms of relative abundance, the main microbial groups were Escherichia*-Shigella*, *Clostridium sensu stricto* 1, Streptococcus, and *Lactobacillus*. Compared to other mammals, the intestinal flora of giant pandas possesses a simple structure and extremely low diversity (9, 10, 36). This may be due to the ability of giant pandas to retain the structural characteristics of the digestive tract of predators and their short rectum, and they do not possess a blind bowel structure. This is somewhat different from the intestinal structure of mice (3, 37). The main phyla *Firmicutes*, *Bacteroides*, and *Actinobacteria* have previously been reported in mice (38, 39). The results of this study were the same but differed in regard to abundance. Concurrently, as the rearing of mice is easier to control, the composition of the intestinal flora was very stable in this study. We observed that the Shannon diversity index of mice was significantly higher than that of the giant pandas, with *Firmicutes*, *Proteobacteria*, and *Bacteroides* being the dominant flora. Interestingly, we observed that the genera Escherichia*-Shigella*, *Clostridium sensu stricto* 1, and Streptococcus were more abundant in giant pandas. These genera help maintain stability in the intestines and intestinal flora and inhibit the production of pathogenic bacteria, and when pandas develop anorexia, we previously observed that the numbers of *Weissella* and Streptococcus in the intestines were decreased (18). Combining these results indicated that the composition of the intestinal flora of giant pandas and mice are different. Therefore, it is inappropriate to study the mechanism of interaction between the intestinal flora and the host of giant pandas directly in mice, and it is necessary to simulate giant panda intestinal flora patterns in mice.

After pseudosterile (antibiotic-treated) mice received fecal microbiota transplantation, the composition and contribution network characteristics of some giant panda gut microbiota were retained; however, they were transformed into new gut microbiota. Our results suggest that the gut microbiota of antibiotic-treated mice is similar to that of giant pandas on the third day after fecal microbiota transplantation. Pseudosterile (antibiotic-treated) mice with intestinal flora are currently an important model for studying animal health and disease in the context of fecal bacterial transplantation (40–42). Compared to germfree mice, antibiotic-treated mice are easier to obtain, raise, and manage, and the cost is lower. Consistent with previous studies, long-term exposure to antibiotics cannot completely eliminate intestinal flora. After antibiotic treatment, the original dominant genus abundance of mice was reduced, but the uniformity and diversity were higher. Additionally, their composition was different from that of untreated mice. However, it is not clear what role the few bacterial families with increased abundance may play. It was previously reported that Klebsiella spp. overgrowth after antibiotic treatment may play an important leading role in the microbiome or may be harmful to animals (43). *Firmicutes* and *Proteobacteria* flora are important carriers of antibiotic resistance, and we observed that the relative abundance of Escherichia, Staphylococcus, and Pseudomonas increased in antibiotic-treated mice (44–48). After fecal microbiota transplantation, the relative abundance of Streptococcus, *Lachnoclostridium*, *Stenotrophomonas*, *Ochrobactrum*, and *Enterococcus* maintained a consistent trend with that of giant pandas on day 3. Conversely, different antibiotic treatment programs influence will impact the effect of fecal bacterial transplantation. Studies have reported that longer antibiotic removal cycles can increase the colonization rate of alien flora. In contrast, the frequency and dosage of FMT also affect the similarity between the intestinal flora of the recipient animal and the intestinal flora of the donor (49, 50). Additionally, the drug-resistant flora in the AT group were partially reduced in the AM group, but the reason for this reduction was unclear. Research by Hu et al. has demonstrated that FMT can eliminate some antibiotic-resistant bacteria, and the special interaction between foreign bacteria and drug-resistant bacteria is also an explanation that cannot be ignored (41, 51). In general, the proportion of giant panda intestinal flora among the intestinal flora of mice treated with antibiotics in this study was not high, and this was consistent with the results of the FMT study using antibiotic mouse models. Existing studies have reported that the choice of recipient animals, the type and treatment of antibiotics, and the dose of donor inoculation are key factors for improving the success rate of FMT colonization in mice treated with antibiotics.

The fecal microbiota from giant pandas was successfully colonized in germfree mice, and there was no significant difference in alpha diversity (only the Shannon diversity on day 21 was significant). Germfree mice after transplantation stabilized after 2 weeks. It is well established that intestinal flora are elastic and can recover within 2 to 4 weeks after disturbing the composition of the flora in ordinary mice, while germfree mice can stabilize at 3 to 9 weeks after transplantation. However, this is not the gold standard (52, 53). In this study, germfree mice exhibited intestinal flora that was dominated by *Firmicutes*. The early bacterial communities formed during the colonization of mammals are primarily composed of the facultative anaerobes Escherichia and *Enterococcus*. The abundance of the *Proteobacteria* phylum decreased or even became undetectable with colonization time, and *Firmicutes* dominated the final colony community (54). Multiple fecal microbiota transplantations allowed for more colonization of the giant panda gut microbiota in the first 3 days, and this accelerated the stability of *Proteobacteria* and *Firmicutes*. Germfree mice exhibited different abundances of *Lactobacillus*, *Lachnoclostridium*, *Turicibacter*, *Stenotrophomonas*, and *Blautia* in the test results after 2 weeks, and these were originally at a low undetectable limit level in the giant panda fecal microbiota. The colonization and high-abundance characteristics of *Lachnospiriaceae* and *Ruminococcaceae* are regarded as health markers, particularly for certain Clostridoides difficile infections (54–56). The composition of fecal microorganisms and the contents of the cecum were similar. The cecum of germfree mice is primarily colonized with anaerobic bacteria related to sugar degradation, such as *Prevotellaceae*, *Lachonospiraceae*, *Ruminococcaceae*, and *Bacteriodetes* (55). These fecal bacteria are easier to colonize and detect in the giant panda after transplantation.

The interaction between floras shapes different ecological communities in the same environment, and the interaction between different bacteria is very complex. The interaction is not only limited to symbiosis and antagonism but also plays a very important role in ecological communities and the host environment (57–59). In this study, different intestinal environments influenced the intestinal flora of giant pandas, and germfree and pseudosterile mice exhibited different characteristics of flora co-occurrence networks. Individuals with unique interactions between intestinal microorganisms are crucial to the stability of healthy microbial communities (58, 60). Microorganisms with dense communication connections are of great significance in an intestinal flora communication co-occurrence network. Intestinal health is an important issue for giant panda survival, and this has been confirmed in a previous retrospective analysis of the causes of death in pandas (5, 14). The species contained within the module of co-occurrence networks that are defined as key species represent the main features of the gut microbiota co-occurrence network. Microorganisms with intensive communication are often the core microorganisms in flora. Although they exhibit different abundances at different time points, they run through the entire life cycle of the host (61). The disappearance of such microorganisms can cause flora imbalance and lead to health problems (62, 63). The peripheral flora characterized as NA here could not represent the core flora in the intestine, and the core is likely to change fundamentally with changes of external environment and the passage of time. The key species and communication links contained in the module species of the bacterial co-occurrence network of germfree mice were consistent with the key species communication characteristics of giant pandas but were accompanied by a decrease in nodes and weakened communication. We noticed that some fecal microbiota of giant pandas were lost during transplantation. This was consistent with human flora-associated (HFA)-related research reports that have indicated that the difference in host genetic factors (for non-germfree animals, selective colonization resistance imposed by the host’s natural microbiota) causes only a portion of the microorganisms to colonize or that host phenotype is abnormal after the donor microorganism is colonized (64–66). Studies have reported that the flora derived from the contents of different intestinal segments of the donor is more likely to establish a flora in the corresponding intestinal segment of the new host during FMT and to induce more colonization of this segment of foreign microorganisms (54, 55). It cannot be ignored that after fecal bacteria transplantation, mice were fed sterilized feed that was different from the staple food of giant pandas. It has been reported that the differences in the composition of the gut microbiota between human individuals may be partially caused by dietary differences, and even the significant changes in the composition of the intestinal flora of HFA mice occur only within a few hours of diet change (67–69). The peak map based on core flora analysis revealed changes in key species in the module of in the mouse internal network. These key species classifications were well maintained in germfree mice. Particularly for a single fecal microbiota transplantation, the key species classification statistics from giant pandas were close to the donors throughout the test cycle and were clustered with giant pandas in the same cluster. Similar to the research report of synthetic bacterial community Oligo-Mouse-Microbiota (OMM) mimicking flora related to fecal bacteria transplantation, multiple inoculations of germfree mice with OMM microbiota can ensure the colonization of microorganisms that are not easy to colonize during a single inoculation but lead to more microbiota changes (70). Unlike germfree mice, pseudosterile mice flora that were only similar to the giant panda fecal microbiota network characteristics and key taxonomic species on day 3. The giant panda fecal microbiota was transplanted into pseudosterile mice and germfree mice, and the intestinal flora of the mice gradually exhibited the characteristics of the giant panda fecal microbiota communication co-occurrence network. The network reproduction effect of germfree mice was better than was that of pseudosterile mice, and germfree mice with single fecal bacteria transplantation exhibited the best effect.

High-throughput technology confirmed that the giant panda fecal microbiota can colonize the intestine of mice. In terms of microbiota, germfree mice after fecal microbiota transplantation were similar to those of giant pandas, and the original intestinal flora network characteristics of giant pandas were well retained and simulated. This is the key information for the establishment and development of flora-associated mouse models of giant pandas. However, to establish an animal model that can simulate the intestinal system of giant pandas, it is still necessary to study how these floras transplanted into the model affect the metabolism and immune characteristics of mouse intestines after interacting with the mouse intestine. Unfortunately, these contents were not covered in this study; however, these studies not only contribute to the complete evaluation of the giant panda flora-associated mouse model but may also lead to some breakthroughs in the study of the interaction between the intestinal flora and the host. Currently, the mouse model of intestinal microbial transplantation has become the most effective choice to study the causal relationship between microorganisms and certain diseases and to verify the preclinical concept that may effectively intervene in the treatment. We expect this study to provide a new and potential alternative animal model for the study of digestive system diseases in giant pandas. The intestinal microbial-derived mouse model constructed by transplanting intestinal microorganisms into mice will help to effectively conduct research on the effect of intestinal microbial intervention in giant pandas, to analyze the causal relationship between microbial interactions, and to achieve the purpose of verifying the hypotheses regarding related mechanisms of disease occurrence.

### Conclusion.

The diversity, species composition and abundance, and bacterial network characteristics of giant panda and mouse intestinal flora are different. After fecal microbiota transplantation, the fecal microbiota of giant pandas colonized mice, and these species exhibited different trends in mice. The key species in the gut microbiota communication co-occurrence network exhibited the same pattern as that in giant pandas in germfree mice and formed a similar co-occurrence network communication mode. This co-occurrence network communication mode was more prominent in germfree mice, particularly those with single fecal microbiota transplantation, and it was more homologous with giant pandas (unlike mice). The ability of pseudosterile mice to maintain the co-occurrence network communication mode of intestinal flora and its key species in giant pandas is weaker than that of germfree mice. This study is helpful for the establishment and exploration of a mouse model of intestinal flora of giant pandas and provides insights for the application of this model in the study of intestinal flora and diseases in giant pandas in the future.

## MATERIALS AND METHODS

### Ethics statement.

All animal procedures were performed in strict accordance with the legislation for care and use of laboratory animals of the People’s Republic of China and approved by the Animal Ethics Committee of Sichuan Agricultural University (approval number SYXKchuan2019-187).

### Giant panda feces sample collection.

According to the donor identification and selection criteria described by Hamilton et al., 10 healthy adult pandas (Table 1) were selected from the Chengdu Research Base of Giant Panda Breeding (CRBGPB) (71). They had not received any antibiotics or probiotics in the 6 months prior to the collection of feces and had not suffered from diarrhea or other intestinal diseases during the same period. Ten fecal samples (100 g each) were collected from each giant panda. After the giant pandas had finished defecation, a disposable sampling spoon was used to place the stool sample into a 50-mL centrifuge tube. The samples were then immediately placed into a liquid nitrogen tank, and all samples were transported to the laboratory for storage in a −80°C freezer. All giant panda fecal samples were collected between 14:30 and 16:30 on the same day.

**TABLE 1:** Basic information for the 10 giant pandas

Name	Pedigree no.	Age (yrs, by 2022)	Gender	Date of birth (yr-mo-day)
Qi Zhen	490	23	Female	1999-09-04
Jing Jing	598	16	Female	2005-08-30
Ya Zai	637	16	Female	2006-08-19
Si Yuan	593	17	Female	2004-10-22
Xiang Bing	665	14	Female	2007-06-30
Ya Li	762	13	Female	2009-07-19
Mei Bang	737	14	Female	2008-09-13
Xing Bang	614	17	Male	2005-08-23
Fu Fu	532	21	Male	2001-08-25
Mei Lan	649	16	Male	2006-09-06

### Mice.

**FIG 8:**
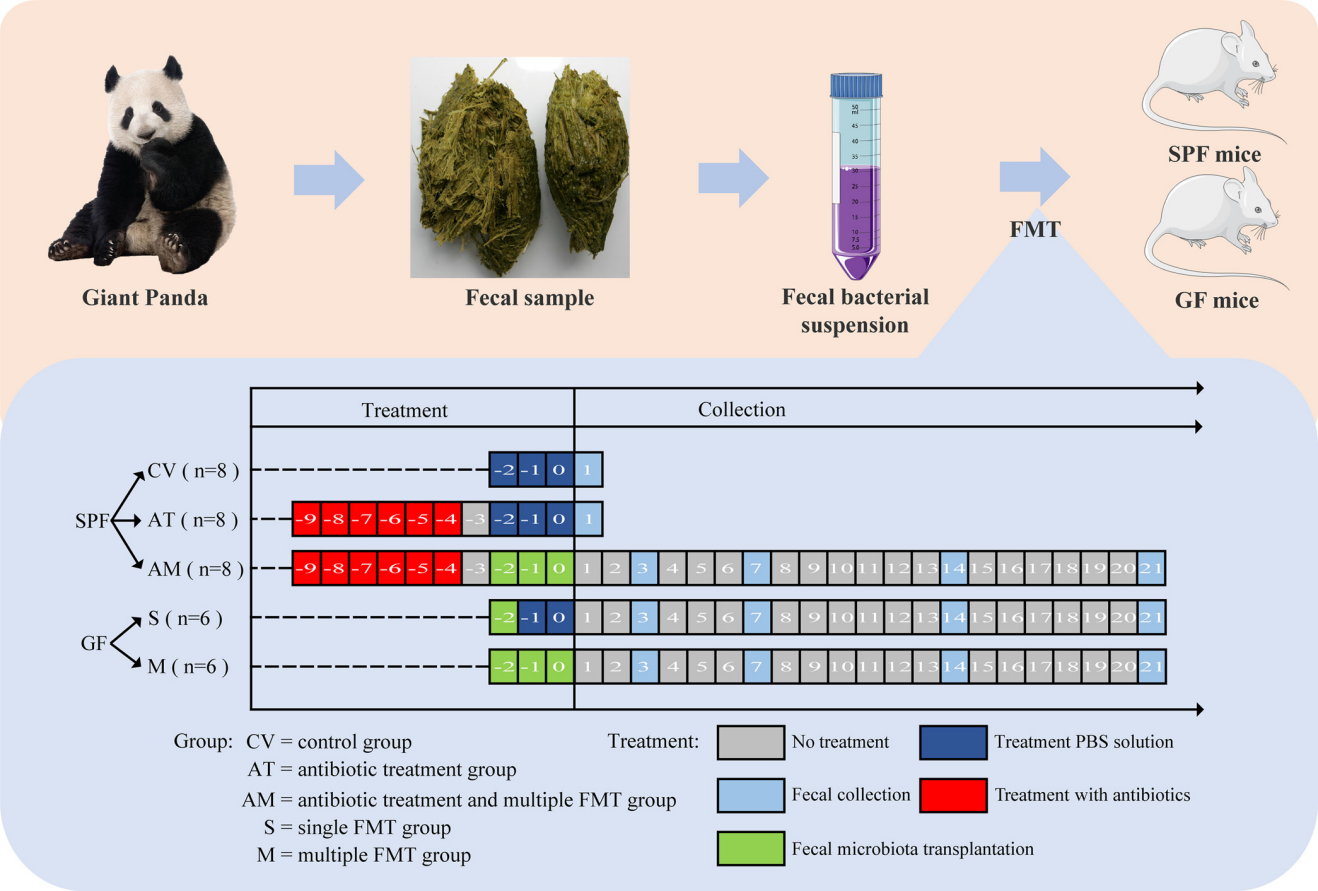
Experimental group design for mice receiving fecal bacteria transplantation. SPF mice were randomly divided into three groups: CV, AT, and AM. For CV mice, 0.2 mL PBS was inoculated by gavage each time for three consecutive days. AT and AM were treated with antibiotics, 100 mg/kg of neomycin sulfate and streptomycin, orally administered twice a day for 6 days. AM was followed by fecal bacteria transplantation, 0.2 mL of a donor bacteria suspension was inoculated by gavage each time for three consecutive days. Germfree (GF) mice were randomly divided into two groups of M and S. Both M and S underwent fecal microbiota transplantation. A 0.2-mL aliquot of donor bacteria suspension was inoculated by gavage each time for three consecutive days. For mice in group S, 0.2 mL of donor bacteria suspension was inoculated by gavage on the first day and 0.2 mL of PBS was inoculated by gavage each time on the second and third days.

All mice used for this study were KM genotypes and included specific-pathogen-free (SPF) and germfree mice. The mice were divided into five groups (Fig. 8). SPF mice were received at 2 weeks of age and were randomly divided into two groups, with each group containing eight mice with the same number of males and females. The three treatment groups were as follows: (i) control group (CV), (ii) antibiotic treatment group (AT), and (iii) antibiotic treatment and multiple fecal microbiota transplantation group (AM). AT and AM group mice were prepared according to the method of Liu et al., and 100 mg/kg of neomycin sulfate and streptomycin were orally administered twice each day for 6 days (72). No antibiotics were administered on the seventh day when the mice reached 3 weeks of age. Three-week-old germfree mice were randomly divided into two groups, with each group containing six mice that were reared in a sterile isolator. (iv) For the single fecal microbiota transplantation group (S) and (v) the multiple fecal microbiota transplantation group (M), the mice were provided with AIN93 standard feed that was sterilized by Co-60 gamma rays (50 KGy) and with drinking water that was sterilized by autoclaving (121°C, 60 min). Mice were housed under a 12-h light-dark cycle at 23 ± 2°C and were allowed to eat and drink freely. The litter, cages, and drinking bottles were sterilized by autoclaving.

### Donor bacteria suspension.

To obtain the fecal microbiota from giant pandas, stool samples from each giant panda were processed separately and then mixed for fecal inoculation. Ten grams of each fecal sample was dispersed into 20 mL of sterile phosphate buffer and filtered through gauze. The residue was washed with 20 mL of sterile phosphate buffer and filtered again to collect the liquid, and the two filtrates were combined into one portion. A total of 20 mL from each of the 10 liquids was obtained, and they were mixed to form a 200-mL suspension of donor bacteria. All operations were rapidly performed under sterile anaerobic conditions. According to the method of Rawls et al., the donor bacterial suspension was inoculated on a brain heart infusion agar plate and cultured at 37°C under anaerobic and aerobic conditions for 2 days, and the viable bacteria were then counted to estimate the approximate bacteria counts per milliliter of specimen (73). The bacterial suspension was diluted to 5 × 10^4^ CFU/mL.

### Microbiota colonization and housing.

As presented in Fig. 8, for mice in the CV and AT group, animals were inoculated by gavage with 0.2 mL phosphate-buffered saline (PBS) each day for three consecutive days. For the AM group, 0.2 mL of the donor bacteria suspension was inoculated by gavage each day for three consecutive days. For mice in group S, 0.2 mL of the donor bacterial suspension was inoculated by gavage on the first day, and 0.2 mL of PBS buffer was inoculated by gavage on the second and third days. For mice in group M, 0.2 mL of the donor bacterial suspension was inoculated by gavage each day for three consecutive days. During the test, clinical symptoms were observed and recorded daily.

### Mouse fecal samples collected.

As presented in Fig. 8, on the first day after the end of PBS treatment, fecal samples were collected from mice in the CV and AT groups. We collected fecal samples from AM, S, and M mice on days 3, 7, 14, and 21 after fecal microbiota transplantation (the day of the end of fecal bacteria transplantation was recorded as day 0). Fecal samples were collected using warm and humid cotton balls to stimulate and lightly press the lower abdomen of mice and were immediately stored in a −80°C freezer for later use.

### DNA isolation and 16S rRNA amplicon sequencing.

Total genomic DNA samples were extracted using the EZNA stool DNA Kit (Omega BioBio-Tek, Norcross, GA, USA) following the manufacturer’s instructions and stored at −20°C prior to further analysis. The quantity and quality of extracted DNA were measured using a NanoDrop NC2000 spectrophotometer (Thermo Fisher Scientific, Waltham, MA, USA) and agarose gel electrophoresis, respectively. PCR amplification of the bacterial 16S rRNA gene V3-V4 region was performed using the forward primer 338F (5′-ACTCCTACGGGAGGCAGCA-3′) and reverse primer 806R (5′-GGACTACHVGGGTWTCTAAT-3′). Sample-specific 7-bp barcodes were incorporated into the primers for sequencing. The PCR components contained 5 μL of buffer (5×), 0.25 μL of Fast *Pfu* DNA polymerase (5 U/μL), 2 μL (2.5 mM) of deoxynucleoside triphosphates, 1 μL (10 μM) of each forward and reverse primer, 1 μL of DNA template, and 14.75 μL of double-distilled H2O. Thermal cycling consisted of initial denaturation at 98°C for 5 min that was followed by 25 cycles consisting of denaturation at 98°C for 30 s, annealing at 53°C for 30 s, and extension at 72°C for 45 s, with a final extension of 5 min at 72°C. PCR amplicons were using Vazyme VAHTSTM DNA Clean beads (Vazyme, Nanjing, China) and quantified using the Quant-iT PicoGreen dsDNA assay kit (Invitrogen, Carlsbad, CA, USA). After the individual quantification step, amplicons were pooled in equal amounts, and pair-end 2 × 250-bp sequencing was performed using the Illumina MiSeq platform at Shanghai Personal Biotechnology Co., Ltd. (Shanghai, China).

### Sequence analysis.

Microbiome bioinformatics analysis was performed using QIIME2 with slight modifications according to the official tutorials (https://docs.qiime2.org/) (74). Briefly, raw sequence data were demultiplexed using the demux plugin, and this was followed by primer cutting using the cutadapt plugin (75). Sequences were then quality-filtered, denoised, and merged, and chimera were removed using the DADA2 plugin (76). Non-singleton ASVs were aligned with mafft and used to construct a phylogeny using fasttree2 (77, 78). All samples were rarefied to 14,419 sequences for the rarefaction of microbiota and the observed ASV richness (79, 80). Taxonomy was assigned to ASVs using the classify-sklearn naïve Bayes taxonomy classifier in the feature-classifier plugin against the SILVA release 138 database (81, 82).

### Bioinformatics and statistical analysis.

In-depth sequence analyses were primarily performed using the QIIME2 and R packages (v3.2.0). Alpha diversity indices, such as the richness estimator and Shannon diversity index, were calculated using the even abundance table and visualized as boxplots. We used the trimmed average M (TMM) method in edgeR package to standardize the number of filtered ASV sequences (83). Beta diversity analysis was performed to investigate the structural variation of microbial communities across samples using Bray-Curtis metrics and was visualized via PCoA (84, 85). The significance of the differentiation of microbiota structure among the groups was assessed by ADONIS using QIIME2 (86, 87). Taxonomic composition and abundance were visualized as stacked histograms using R software. We have only presented the distribution of phylum and genus levels with relative abundances of ≥1% in different groups, and those with relative abundances of >1% were characterized as “Others.” Subsequently, we tested for significant alterations in microbial taxa (including phyla and genera) between each group using the Wilcoxon test. LEfSe was used to detect differentially abundant taxa across groups (88). There was a linear discriminant analysis with the standard test for statistical significance, including a Kruskal-Wallis test and Wilcoxon tests. Microbial taxa whose *P* value (factorial Kruskal-Wallis test) and the threshold for discriminative features (logarithmic LDA score) were calculated for each group at a *P* level of <0.05 and LDA score of >4.0, respectively.

Indicator species based on significant (*P* < 0.01) results were displayed in the bipartite network, and the correlation between ASV and one or more different ASVs was labeled. The bipartite network was built from a Fruchterman Reingold layout with 10^4^ permutations. Through the analysis of indicator species, we determined the iconic between the significant (*P* < 0.01) changes in ASV and different animals (giant panda, SPF mice, pseudosterile mice) to determine the specific intestinal flora patterns that play an important role in different animal species (89). SparCC analysis was performed between the ASVs. The pseudocount value for SparCC was set at 0.05. The cutoff of correlation coefficients was determined as 0.3 using random matrix theory-based methods as implemented in R. Based on the correlation coefficients, we constructed a co-occurrence network with nodes representing ASVs and edges representing correlations between these ASVs. The network was visualized using the R package igraph. The intersection analysis of the indicator species results with ASVs included in the SparCC co-occurrence network retained a network of indicator species and their associated species shared by both. We used a greedy optimized modular algorithm to identify and visualize community modules related to indicator species in co-occurrence networks and extracted all species in community modules for statistical analysis (90). We defined the ASVs contained in the module as “key species” and ASVs that did not belong to any module as “peripheral species.” Finally, species annotation and statistical classification analyses were performed on the key species in the module. A peak-mountain diagram was used to observe the changing trend of the key species in each group. A heatmap based on key species was used to reflect the similarity in the trends of key species in each group.

### Statistics.

Summary statistics of data are reported using means with standard errors. All error bars represent the standard error of the mean. Wilcoxon tests were used to compare the differences of richness and Shannon diversity index between groups. Subsequently, we tested for the significant alterations of microbial taxa, including phylum and genus, between each group using a pairwise Wilcoxon test. There was linear discriminate analysis with the standard tests for statistical significance, including the Kruskal-Wallis test and Wilcoxon test.

### Data availability.

The complete experimental data set for the giant pandas and mouse fecal samples has been deposited in the Sequence Read Archive database under accession number PRJNA843223.
