# Supplementary material for: Dynamic Monitoring of Changes in Fecal Flora of Giant Pandas in Mice: Co-Occurrence Network Reconstruction
Source: Microbiol Spectr. 2022 Dec 6;11(2):e01991-22. doi: 10.1128/spectrum.01991-22 (PMC10100740; doi:10.1128/spectrum.01991-22)
Supplement: Supplemental file 1 — Supplemental material. Download spectrum.01991-22-s0001.pdf, PDF file, 1.5 MB [file spectrum.01991-22-s0001.pdf]

Supplemental Material

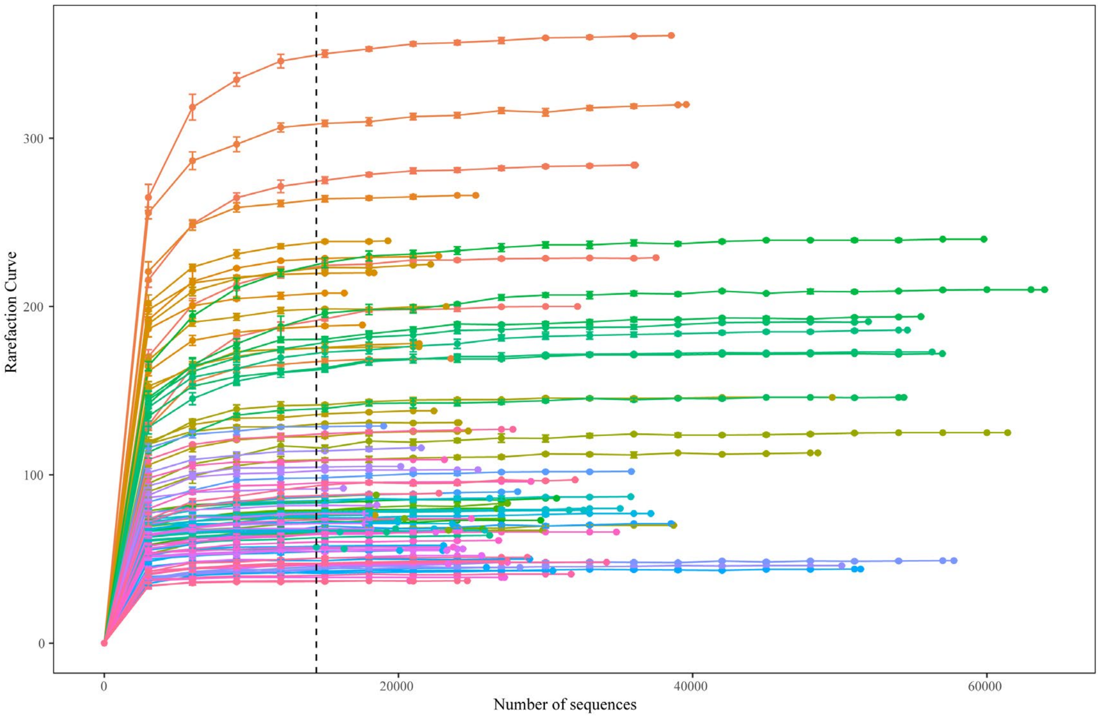

Figure S1: Rarefaction curves of all samples

Rarefaction curves of fecal samples from giant panda, SPF mice, and GF mice. Each line represents a sample, a total of 107 samples. The abscissa represents the number of reads sequenced, and the ordinate represents the number of amplified sequence variants. All samples were rarefied to 14,419 sequences for the follow rarefaction of microbiota and the observed ASV richness.

A.)

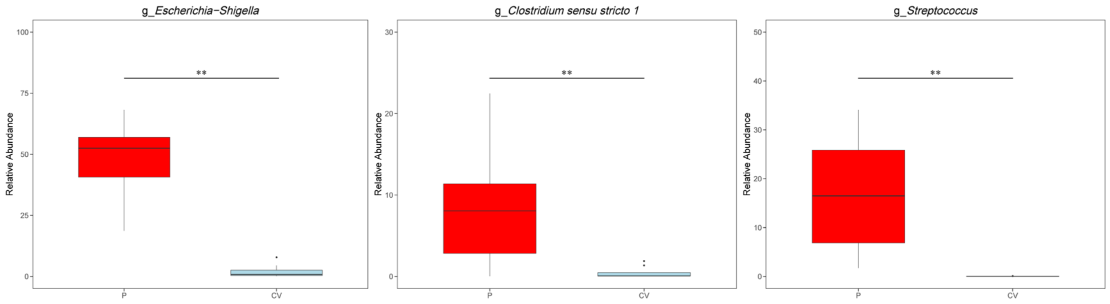

B.)

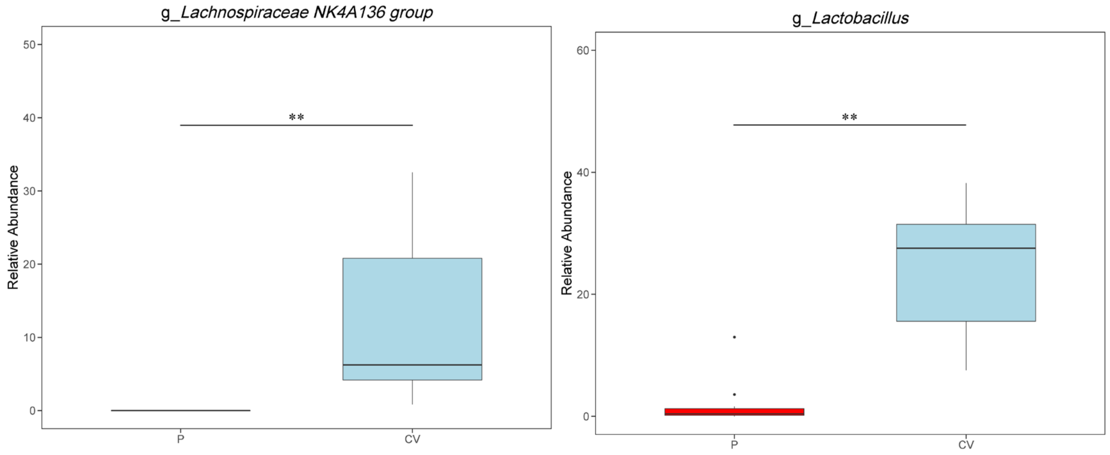

Figure S2: Genus with horizontal abundance differences

Taxa that were significantly differentially represented between groups were examined by linear discriminant analysis coupled with effect size (LEfSe) using the parameters (LDA score = 4). **A.)** Based on LEfSe analysis, *Escherichia-Shigella*, *Clostridium sensu stricto 1* and *Streptococcus* were abundant in giant panda fecal floras compared with mice. **B.)** *Lachnospiraceae NK14A136 group* and *Lactobacillus* were abundant in mice fecal floras compared with a giant panda. Statistical differences were determined by the Kruskal-Wallis test (*, P < 0.05. **, P<0.01). All of the data are expressed as means with standard deviations. P: giant pandas, CV: SPF mice without any treatment.

A.)

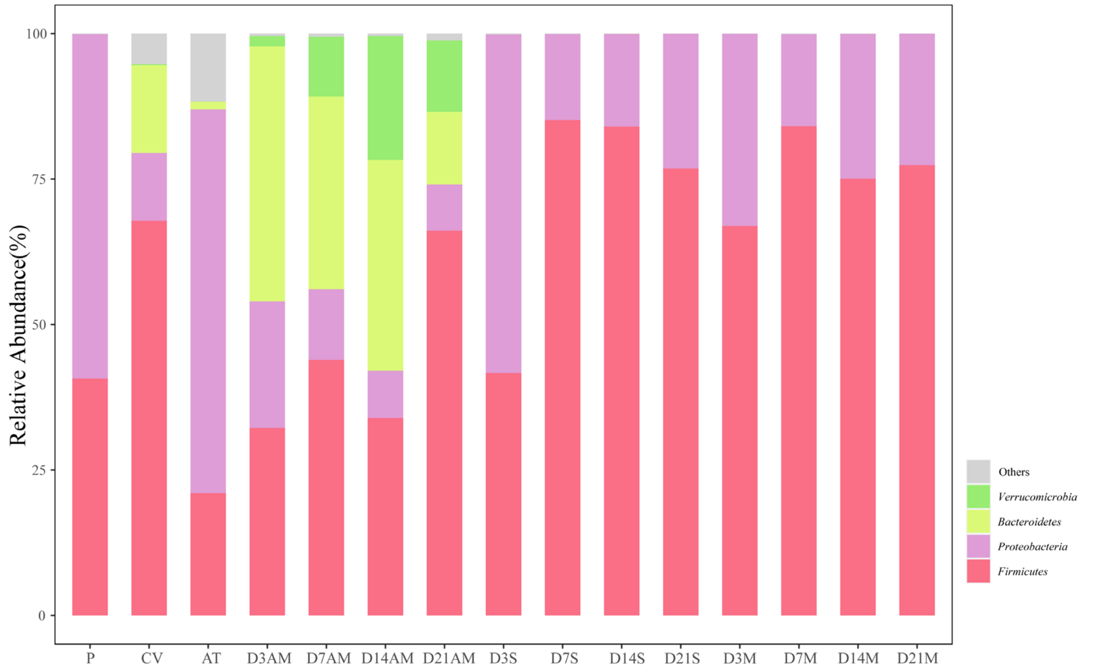

B.)

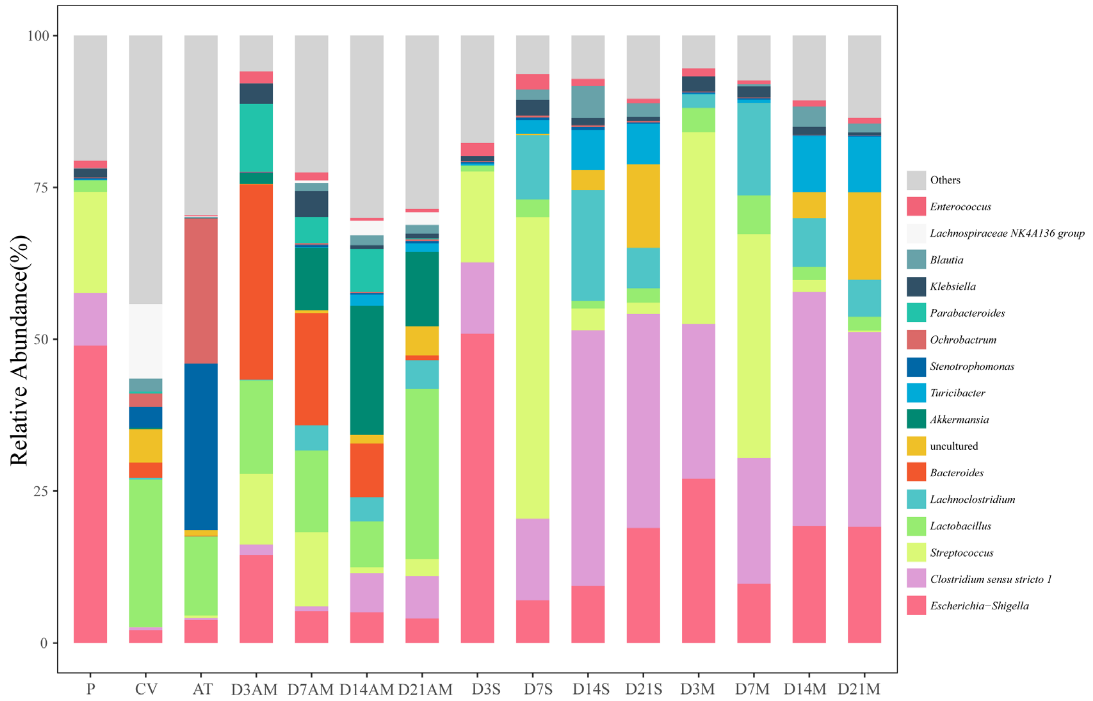

Figure S3: Relative abundance of fecal flora in giant pandas, mice and fecal microbiota transplantation mice.

All sequencing sequences obtained in the experiment were subjected to quality filtering, denoising, merging, and chimera removal, and the samples were flattened to obtain 312,1274 reads, and 2461 ASVs were obtained by clustering. The species annotation of ASVs was performed according to the classifier trained by SILVA SSU 138 database. Distributions of relative abundance of top species at the **A.)** phylum and **B.)** genus level. Each color represents each species. The height of the column represents the abundance of reads(We only present the distribution of phylum and genus levels with relative abundances of greater than or equal to 1% in different groups, and those with relative abundances of less than 1% are characterized as 'other'). P: giant pandas, CV: SPF mice without any treatment, AT: SPF mice treated with antibiotics, AM: multiples gavage with giant panda fecal floras after SPF mice treated with antibiotics, M: germ-free mice multiples gavage with giant panda fecal floras, S: germ-free mice single gavage with giant panda fecal floras.

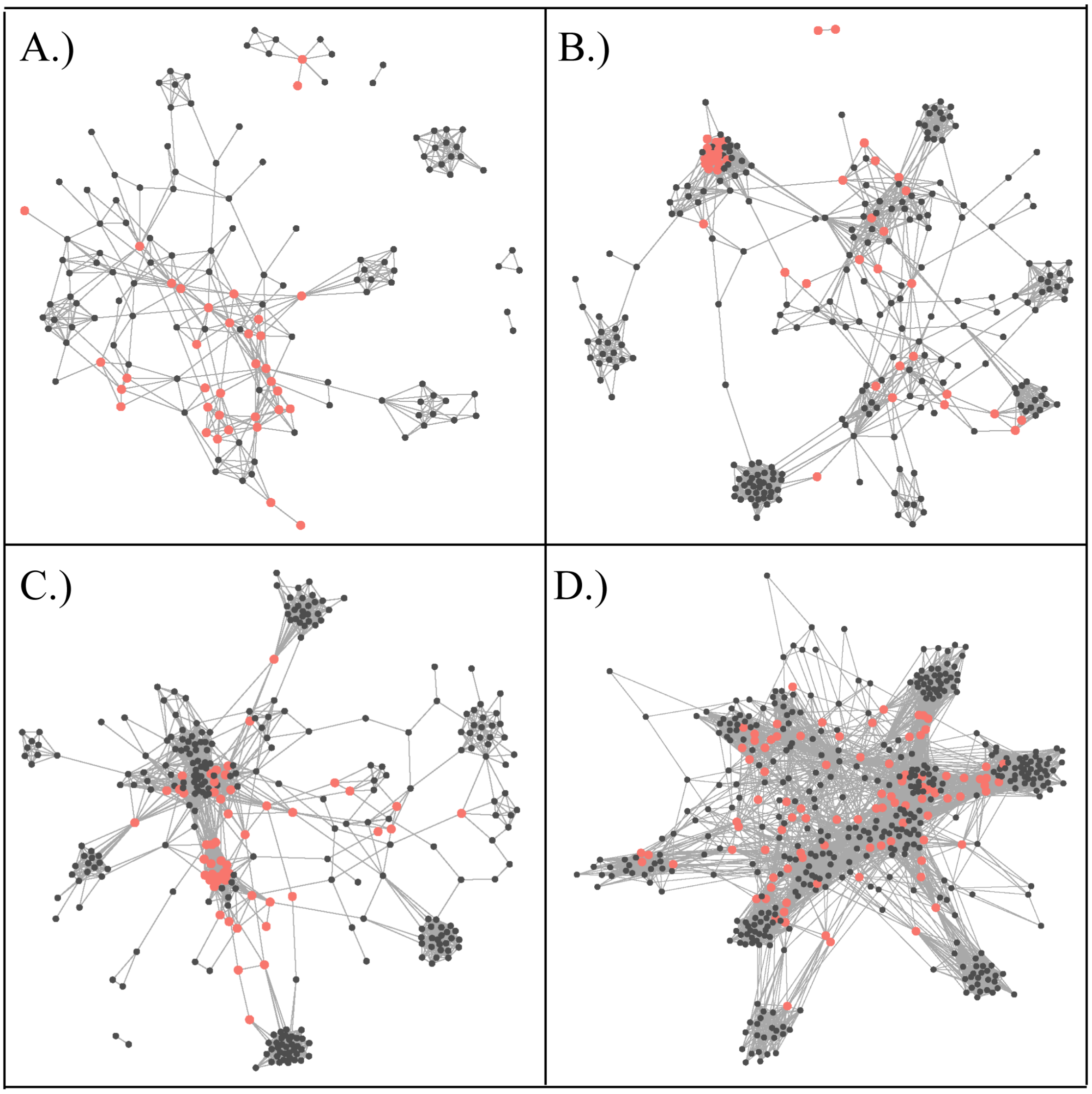

Figure S4: Bacterial community co-occurrence network based on indicator species in pseudo sterile mice (AM group).

Indicator species analysis between P, CV, and AT groups were visualized by the bipartite network, and the corresponding indicator species were obtained. Based on the indicator species of P group, visualize the co-occurrence network of bacteria associated in AT group the with indicator species of P group. **A.)** Co-occurrence networks associated with P group indicator species on the 3 days of AM group, **B,)**, **C.)** and **D.)** Co-occurrence Networks of AM group on 7, 14, and 21 days, respectively. ($r > 0.3$, p

< 0.05; The relationship between ASVs is represented by gray edges; Indicator species ASVs marked in red nodes; ASVs closely related to indicator species are represented by black nodes.)

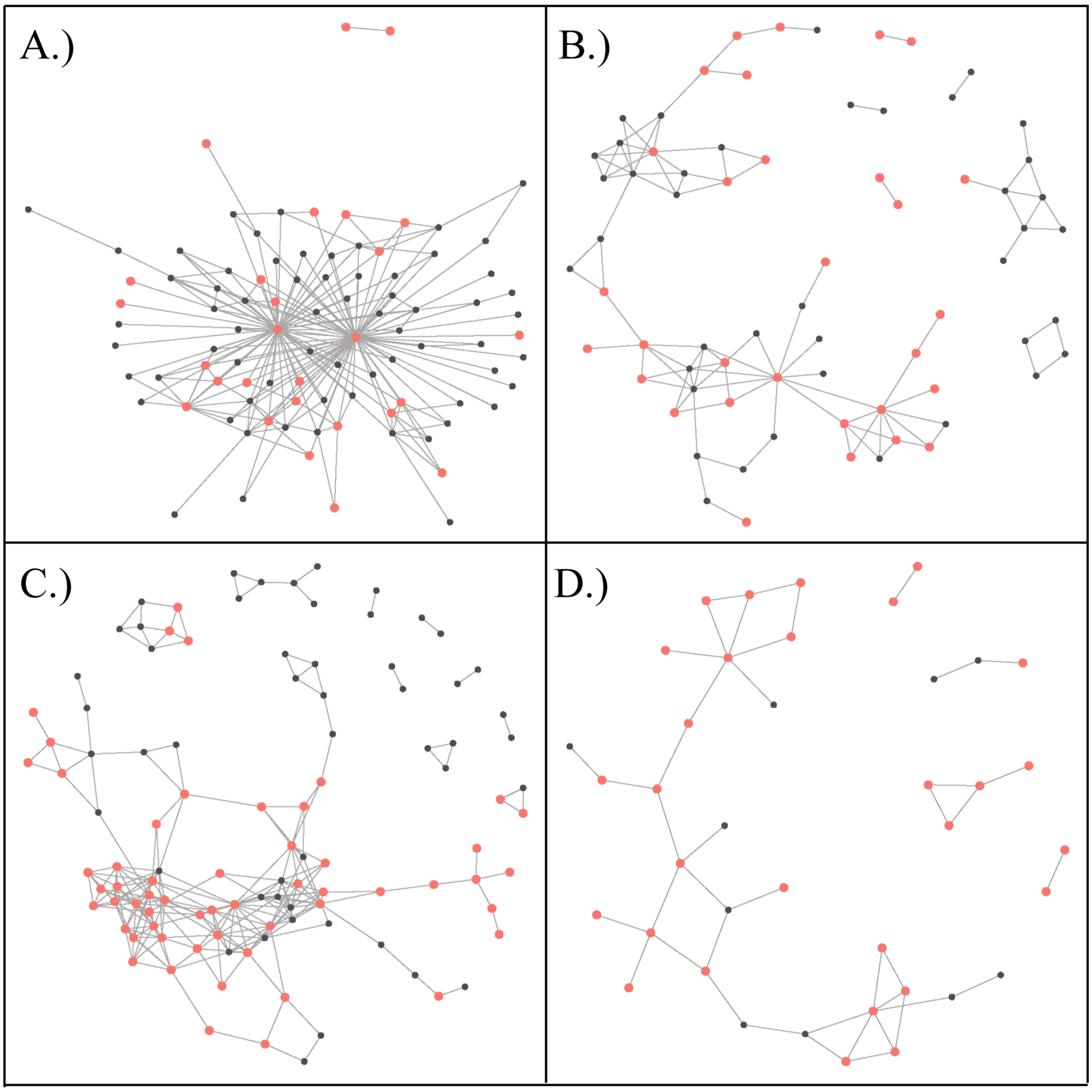

Figure S5: Bacterial community Co-occurrence Network based on indicator species in pseudo sterile mice (M group).

Based on the indicator species of P group, visualize the co-occurrence network of bacteria associated in M group with the indicator species of P group. **A.)** co-occurrence networks associated with P group indicator species on day 3 of M group, **B.)**, **C.)** and **D.)** co-occurrence networks of M group on day 7, 14, and 21, respectively. ($r > 0.3$, $p < 0.05$; The relationship between ASVs is represented by gray edges; Indicator spe-

cies ASVs marked in red nodes; ASVs closely related to indicator species are represented by black nodes.)

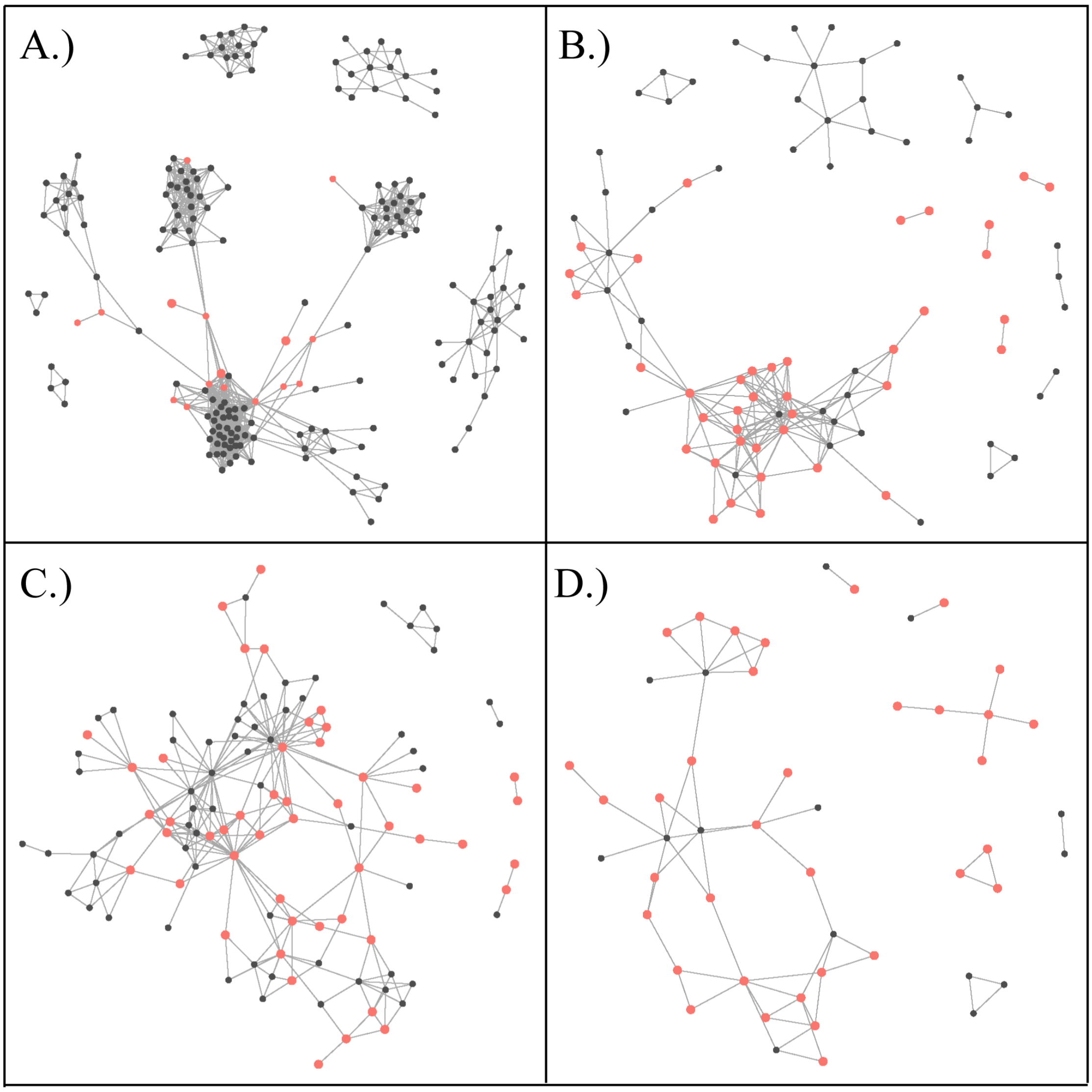

Figure S6: Bacterial community Co-occurrence Network based on indicator species in pseudo sterile mice (S group).

Based on the indicator species of P group, visualize the co-occurrence network of bacteria associated in S group with the indicator species of P group. **A.)** co-occurrence networks associated with P group indicator species on day 3 of S group, **B,), C.)** and **D.)** co-occurrence networks of S group on day 7, 14, and 21, respectively. ($r > 0.3$, $p < 0.05$; The relationship between ASVs is represented by gray edges; Indicator species ASVs marked in red nodes; ASVs closely related to indicator species are represented

by black nodes.)

Table S1: Indicator species and Co-occurrence Network topology parameters

Group	Indicator_species_all	Indicator_species_model	Nodes_num	Edges_num	Average_degree	Average_path_length	Graph_diameter	Clustering_coefficient	Betweenness_centralization	Degree_centralization	Core-correlations_percentage_in_co-occurrence_network
P	28	24	247	1481	11.9919	2.629171	8.605844	0.74206	0.037242	0.211415	0.8919649
CV	142	141	651	10282	31.58833	2.229143	4.093716	0.695703	0.027407	0.052941	1
AT	100	100	1263	74446	117.8876	1.269912	2.271334	0.730041	0.010031	0.088837	1
D3S	18	16	208	1017	9.778846	3.799349	8.936948	0.658397	0.126789	0.121841	0.8652901
D7S	41	39	104	190	3.653846	3.062682	9.216551	0.458771	0.101001	0.129574	0.8421053
D14S	51	48	128	253	3.953125	3.399745	8.475964	0.276025	0.203565	0.149975	0.972332
D21S	62	35	89	64	1.438202	3.393391	6.981682	0.218182	0.047412	0.063202	0.9375
D3M	29	27	107	211	3.943925	2.186919	4.914295	0.072281	0.378106	0.594869	1
D7M	39	30	98	103	2.102041	4.800723	12.53397	0.323204	0.120841	0.091732	0.9417476
D14M	60	56	120	248	4.133333	3.796758	10.72577	0.420037	0.081954	0.116527	0.9435484
D21M	46	29	96	42	0.875	4.848799	11.76783	0.225	0.04033	0.053947	1
D3AM	37	35	155	473	6.103226	3.370143	7.947309	0.565455	0.119503	0.064265	0.5729387
D7AM	41	39	269	1611	11.9777	3.897227	10.28747	0.68312	0.15731	0.078441	0.7951583

D14AM	52	51	326	2435	14.93865	3.775701	8.803335	0.633988	0.177339	0.101727	0.7946612
D21AM	94	94	507	6191	24.42209	2.267309	4.392892	0.559322	0.234511	0.165174	1

77

Table S2: Significance table of multiple comparisons of alpha diversity

Group	p.signif.Richness	p.signif.Rhannon_diversity	Group	p.signif.Richness	p.signif.Rhannon_diversity
P VS CV	**	ns	AT VS D14S	**	*
P VS AT	**	**	AT VS D21S	**	ns
P VS D3S	ns	ns	AT VS D3M	**	**
P VS D7S	ns	ns	AT VS D7M	**	**
P VS D14S	ns	ns	AT VS D14M	**	ns
P VS D21S	ns	**	AT VS D21M	**	ns
P VS D3M	ns	ns	AT VS D3AM	**	ns
P VS D7M	ns	ns	AT VS D7AM	**	ns
P VS D14M	ns	ns	AT VS D14AM	**	ns
P VS D21M	ns	*	AT VS D21AM	*	*
P VS D3AM	ns	**			
P VS D7AM	ns	**	D3S VS D7S	ns	ns
P VS D14AM	*	**	D3S VS D14S	ns	ns
P VS D21AM	**	ns	D3S VS D21S	ns	ns
			D7S VS D14S	*	ns
CV VS AT	ns	**	D7S VS D21S	**	**
CV VS D3S	**	**	D14S VS D21S	ns	ns
CV VS D7S	**	**			
CV VS D14S	**	**	D3M VS D7M	ns	ns
CV VS D21S	**	**	D3M VS D14M	**	ns
CV VS D3M	**	**	D3M VS D21M	*	*

CV VS D7M	**	**	D7M VS D14M	**	ns
CV VS D14M	**	**	D7M VS D21M	ns	*
CV VS D21M	**	**	D14M VS D21M	*	ns
CV VS D3AM	**	**			
CV VS D7AM	**	**	D3AM VS D7AM	*	ns
CV VS D14AM	**	**	D3AM VS D14AM	**	ns
CV VS D21AM	ns	**	D3AM VS D21AM	**	**
			D7AM VS D14AM	ns	ns
AT VS D3S	**	**	D7AM VS D21AM	**	ns
AT VS D7S	**	**	D14AM VS D21AM	**	**

79

Table S3: Significance table of beta diversity distance multiple comparisons

Group AM	p.signif	Group S	p.signif	Group M	p.signif
CV VS P	**	CV VS P	**	CV VS P	**
AT VS P	**	AT VS P	**	AT VS P	**
AT VS CV	**	AT VS CV	**	AT VS CV	**
D3AM VS P	**	D3S VS P	ns	D3M VS P	**
D7AM VS P	**	D7S VS P	**	D7M VS P	**
D14AM VS P	**	D14S VS P	**	D14M VS P	**
D21AM VS P	**	D21S VS P	**	D21M VS P	**
D3AM VS CV	**	D3S VS CV	**	D3M VS CV	**
D7AM VS CV	**	D7S VS CV	**	D7M VS CV	**
D14AM VS CV	**	D14S VS CV	**	D14M VS CV	**
D21AM VS CV	**	D21S VS CV	**	D21M VS CV	**
D3AM VS AT	**	D3S VS AT	**	D3M VS AT	**
D7AM VS AT	**	D7S VS AT	**	D7M VS AT	**
D14AM VS AT	**	D14S VS AT	**	D14M VS AT	**
D21AM VS AT	**	D21S VS AT	**	D21M VS AT	**
